# Amplifying STING Activation and Alleviating Immunosuppression through a Mn^2+^-Based Metal-Organic Framework Nanosystem for Synergistic Cancer Therapy

**DOI:** 10.34133/bmr.0028

**Published:** 2024-05-02

**Authors:** Mingxiao Fang, Jun Zheng, Jingxue Wang, Chenpeng Zheng, Xiaojing Leng, E. Wen, Pan Li, Haitao Ran, Liang Zhang, Zhigang Wang

**Affiliations:** ^1^ State Key Laboratory of Ultrasound in Medicine and Engineering, Institute of Ultrasound Imaging, The Second Affiliated Hospital, Chongqing Medical University, Chongqing 400010, PR China.; ^2^ Chongqing Emergency Medical Center, Chongqing University Central Hospital, Chongqing, 400014, PR China.; ^3^ Ultrasound Department, The First Affiliated Hospital of Chongqing Medical University, Chongqing 400042, PR China.

## Abstract

The field of immunotherapy, particularly immune checkpoint blockade (ICB), holds immense potential in mitigating the progression of cancer. However, the challenges of insufficient tumor antigen production and the immunosuppressive state in the tumor microenvironment substantially impede patients from deriving benefits. In this research, we present a tumor-microenvironment-modulation manganese-based nanosystem, PEG-MnMOF@PTX, aiming to improve the responsiveness of ICB. Under acidic conditions, the released Mn^2+^ accomplishes multiple objectives. It generates toxic hydroxyl radicals (•OH), together with the released paclitaxel (PTX), inducing immunogenic cell death of tumor cells and normalizing tumor blood vessels. Concurrently, it facilitates the in situ generation of oxygen (O_2_) from hydrogen peroxide (H_2_O_2_), ameliorating the microenvironmental immunosuppression and increasing the efficacy of immunotherapy. In addition, this study demonstrates that PEG-MnMOF@PTX can promote the maturation of dendritic cells and augment the infiltration of cytotoxic T lymphocytes through activation of the cyclic guanosine 5′-monophosphate–adenosine 5′-monophosphate synthase (cGAS) and interferon gene stimulator (STING) pathways, namely cGAS–STING pathways, thereby heightening the sensitivity to ICB immunotherapy. The findings of this study present a novel paradigm for the progress in cancer immunotherapy.

## Introduction

The field of immunotherapy, particularly immune checkpoint blockade (ICB), holds great promise as an effective antitumor modality [1,2]. However, the resistance of solid tumors to immunotherapy, characterized by inadequate production of tumor antigens and immunosuppression within the tumor microenvironment (TME), substantially impedes the clinical effectiveness of ICB [3]. Numerous strategies have been implemented to enhance the immune response of ICB, yielding encouraging outcomes. A prevalent approach involves the induction of immunogenic cell death (ICD) to elicit the exposure or release of damage-associated molecular patterns (DAMPs), thereby triggering antigen presentation mediated by antigen-presenting cells (APCs), such as dendritic cells (DCs) [4]. The DCs also play pivotal roles in the stimulator of interferon gene (STING) pathway by recognizing and binding DAMPs. Recent studies have emphasized the crucial role of the cyclic guanosine 5′-monophosphate (GMP)–adenosine 5′-monophosphate (AMP) (cGAMP) synthase (cGAS)–STING pathway in innate immunity, and numerous immunotherapies have been developed on the basis of its activation [5,6]. During this process, damaged double-stranded DNA (dsDNA) in the cytoplasm is released as DAMPs, then captured by DCs, and catalyzed by cGAS to produce cGAMP. The cGAMP, also named as cyclic dinucleotide, then binds to STING dimers, thereby initiating the oligomerization of STING and subsequent activation of TANK-binding kinase 1 (TBK1) [7,8]. The outcome of this process leads to the generation of diverse immunomodulatory signals, particularly type 1 interferon-β (IFN-β), which subsequently facilitates the maturation of DCs and triggers the activation of CD8^+^ T cells [9–11].

The immunosuppressive TME, including hypoxia, nonetheless hampers the antitumor activity of CD8^+^ T cells [12]. Hypoxia is closely linked to the presence of immunosuppressive cells, including tumor-associated macrophages (TAMs), myeloid-derived suppressor cells (MDSCs), and regulatory T cells (T_regs_), which ultimately result in dysfunction of T cells [13]. In addition, prolonged hypoxia in solid tumors triggers the activation of the hypoxia-inducible factor-1α (HIF-1α) pathway, which promotes the formation of a dense extracellular matrix that resists the accumulation of programmed cell death-ligand 1 antibody (αPD-L1) [14]. Therefore, it is crucial to overcome solid tumor hypoxia to revitalize the antitumor activity of cytotoxic T lymphocytes (CTLs) and improve the effectiveness of ICB-mediated immunotherapy [15].

Manganese, one of the essential human trace elements, is crucial for maintaining proper nervous system function [16,17]. In addition, it plays a pivotal role in tumor immunity. Numerous studies have demonstrated that Mn^2+^ directly activates the cGAS–STING pathway, independent of dsDNA, thereby augmenting innate antitumor immunity and sensitizing ICB immunotherapy [18,19]. The high drug loading capacity, adaptable structure, and unique responsiveness to the TME have rendered Mn-based metal-organic frameworks (MnMOFs) a subject of substantial interest in recent years [20,21]. In the acidic milieu of tumors, MnMOFs with peroxidase (POD)-like activity can generate hydroxyl radicals (•OH) from hydrogen peroxide (H_2_O_2_), causing a certain level of cellular demise and thereby inducing ICD [22,23]. In addition, MnMOFs with catalase (CAT)-like activity can produce oxygen (O_2_) from H_2_O_2_, thereby ameliorating the hypoxic TME and enhancing the efficacy of immunotherapy [24]. The immune-enhancing effects of certain chemotherapeutic agents have been demonstrated. Paclitaxel (PTX), a front-line chemotherapeutic agent in various tumor treatments, not only induces ICD but also mitigates tumor hypoxia by normalizing tumor vasculature [25]. Concurrently, the DNA damage resulting from tumor eradication triggers activation of the cGAS–STING pathway, thereby establishing the combined utilization of PTX as a promising chemoimmunotherapeutic strategy.

**Fig. 1.**
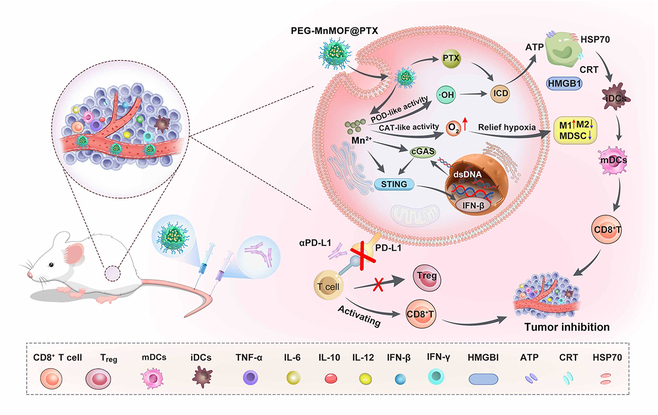
Schematic illustration of the therapeutic strategy of TME-modulated PEG-MnMOF@PTX nanosystem for synergistic cancer therapy.

Given the synergistic interplay between tumor immunotherapy and Mn^2+^, we designed and developed TME-modulated MnMOF nanosystems (PEG-MnMOF@PTX) to enhance the immunotherapeutic efficiency of ICB. The MnMOF was modified with polyethylene glycol (PEG) for enhanced colloidal stability, and the chemotherapeutic drug PTX was integrated with high loading capacity. The detailed mechanism of PEG-MnMOF@PTX action is outlined in Fig. 1. Upon internalization by tumor cells, PEG-MnMOF@PTX undergoes disassembly, leading to the release of Mn^2+^ and PTX. The released Mn^2+^ serves a dual purpose: It converts endogenous H_2_O_2_ to •OH for chemodynamic therapy (CDT) and enhances the hypoxia TME through in situ oxygen generation, subsequently alleviating immunosuppression and improving overall therapeutic efficacy. Meanwhile, the generated Mn^2+^ can be used as a T1-weighted magnetic resonance imaging (MRI) contrast agent for tumor-specific imaging and monitoring. Furthermore, the released PTX, in synergy with Mn^2+^, alleviates tumor hypoxia, normalizes tumor vasculature, heightens the sensitivity of DNA sensors cGAS and STING, and promotes IFN production, thereby reinforcing the antitumor immune response of ICB. The present study has shown a remarkable suppression of tumor growth in mice through the synergistic administration of Mn^2+^ and PTX with αPD-L1. The innovatively engineered PEG-MnMOF@PTX delivery system substantially enhances tumor responsiveness to ICB treatments by boosting tumor immunogenicity and reshaping the immunosuppressive TME, thereby anticipating improved clinical outcomes.

## Materials and Methods

### Materials and reagents

All chemicals, including manganese tetrahydrate (>99.9%), 2,5-dihydroxyterephthalic acid (DHTP; > 98%), *N*,*N*-dimethylformamide (DMF), H_2_O_2_ (30%), sodium bicarbonate (NaHCO_3_) was purchased from Sigma-Aldrich (USA). PTX (99%), 3,3′,5,5′-tetramethylbenzidine (TMB), and glutathione (GSH) were purchased from Shanghai Macklin Biochemical Co. Ltd. (China). Dithionitrobenzoic acid was purchased from Aladdin Reagent Co. Ltd. (China). All chemicals were of analytical grade and used as received.

### Characterization

Transmission electron microscopy (TEM) images were captured by a Talos F200X G2 (Carl Zeiss SUPER-X, Germany). Scanning electron microscope (SEM) images were recorded on a GeminiSEM 300 (Carl Zeiss SUPRA 55, Germany). Size and zeta potential were analyzed by Malvern zetasizer (ZEN3690, UK). The x-ray diffraction (XRD) pattern was recorded on Ultma IV (Rigaku, Japan) at 45 kV and 200 mA. X-ray photoelectron spectroscopy (XPS) was carried out on K-Alpha (Thermo Fisher Scientific, USA). Fourier transform infrared spectrometry spectra were obtained by a Vertex 70 (Bruker, Germany). Thermogravimetric (TG) analysis was performed on a star simultaneous thermal analyzer (Mettler, Swiss). Surface area tested by surface area and porosity analyzer (Micromeritics ASAP 2460, USA). High-performance liquid chromatography was recorded on an UltiMate 3000 RS (Thermo Fisher Scientific, USA).

### Cell lines and animals

The colorectal cancer cell line CT26 cell was purchased from Shanghai Zhongqiao Xinzhou Biotechnology Co. Ltd. (Shanghai, China). Male BALB/c mice (6 to 8 weeks old) were purchased from Chongqing Medical University (Chongqing, China). All mice were housed in a dedicated pathogen-free facility at Chongqing Medical University with a 12-h:12-h light/dark cycle. All animal experiments were conducted and approved by the Animal Ethics Committee of Chongqing Medical University.

### Synthesis of MnMOF

MnMOF nanosystem were synthesized by a self-assembly. Manganese acetate (1.3 mmol) and DHTP (0.5 mmol) were dissolved in 5 and 2.648 ml of DMF, respectively (the molar ratio of the linker to metal = 2.6:1). The DHTP solution was then added into manganese acetate solution in a dropwise manner under vigorous stirring at room temperature. After stirring for 20 h, the resultant was centrifuged with DMF to remove metal ions. It was then washed with methanol and centrifuged twice to remove organic matter [21].

### Synthesis of PEG-MnMOF@PTX

To synthesize PEGylated MnMOF nanosystems, MnMOF was dispersed in water and mixed with phosphate-group-terminated PEG at a PEG:MnMOF mass ratio of 2:1. The mixture was sonicated for 40 min at room temperature and then centrifuged to remove excess PEG. PTX was subsequently dispersed in methanol and mixed with the PEG-MnMOF suspension at a 1:1 mass ratio. After mixing for 24 h under continuous agitation, the resulting product was washed with water and centrifuged twice.

### In vitro multienzyme-like activity

The chromogenic TMB assay was conducted to monitor the interaction between PEG-MnMOF and H_2_O_2_. In detail, a solution of pH 6.5 was prepared with HAc/NaAc in the presence of NaHCO_3_ (25 mM) and mixed with TMB (1 mM), PEG-MnMOF (50 μg·ml^−1^) and H_2_O_2_ (0, 1.25, 2.5, 5, and 10 mM) at 37 °C for 10 h. The absorbance at 652 nm was then observed by ultraviolet-visible (UV-vis) spectra, and the solution in the well was transferred to a tube to be photographed.

We utilized a dissolved oxygen meter (PreSens, Germany) to assess the oxygen production capacity of PEG-MnMOF. In a 4-ml buffer solution (pH 6.5), H_2_O_2_ solution (5 mM) was mixed with 5 μl of PEG-MnMOF (0, 1, 2, 3, and 4 mg·ml^−1^) at 37 °C. Then, the date of changes was observed on the dissolved oxygen meter. In weakly acidic environment, Mn^2+^ successfully catalyzes H_2_O_2_ to oxygen as its concentration increases.

H_2_O_2_ consumption was evaluated by using a H_2_O_2_ kit (Solarbio, China). Briefly, 10 μl of PEG-MnMOF (1 mg·ml^−1^) and 40 μl of H_2_O_2_ solution (2 mM) were added to 200 μl of acetone. Then, 25 μl of acidic Ti(SO_4_)_2_ solution and 50 μl of ammonium hydroxide were added to the above solution and centrifuged for 10 min (4,000*g*). The resulting precipitate was dissolved in H_2_SO_4_ solution. The absorbance of the final solution at 415 nm was measured by UV-vis spectroscopy.

### In vitro cell experiments

For cellular uptake of PEG-MnMOF, CT26 cells were seeded in a confocal dish at a density of 1 ×10^5^ cells per dish and cultured for 24 h. The medium was then replaced with fresh medium containing 1,1′-dioctadecyl-3,3,3′,3′-tetramethylindocarbocyanine perchlorate (DiI)-labeled PEG-MnMOF [PEG-MnMOF (12.5 μg·ml^−1^) and PTX (2.63 μg·ml^−1^)] at different time points (0, 3, 6, 12, and 24 h), after which all samples were washed and fixed with 4% cold paraformaldehyde before confocal laser scanning microscope (CLSM) observation. A quantitative fractionation analysis was also performed using flow cytometry (FCM).

For in vitro cytotoxicity assays, CT26 cells were seeded in 96-well plates (1 × 10^4^ cells per well) and incubated in Dulbecco’s modified Eagle’s medium containing 10% fetal bovine serum and 1% antibiotics at 37 °C for 24 h. Then, the cell was coincubated with PEG-MnMOF (0, 6.25, 12.5, 25, and 50 μg·ml^−1^), PTX (0, 1.31, 2.63, 5.25, and 10.5 μg·ml^−1^), or PEG-MnMOF@PTX [PEG-MnMOF (0, 6.25, 12.5, 25, and 50 μg·ml^−1^) and PTX (0, 1.31, 2.63, 5.25, and 10.5 μg·ml^−1^)] for 24 h, respectively. Finally, culture medium containing 10% of cell counting kit-8 (CCK-8) was added to each well to analyze the cell viability. After incubation at 37 °C for another 1 h, the absorbance at 450 nm of each well was obtained by a microplate reader.

For in vitro reactive oxygen species (ROS) measurement, 2′,7′-dichlorodihydrofluorescein diacetate (DCFH-DA) (Beyotime, China) was used as an ROS probe for detecting the intracellular generation of ROS. CT26 cells were seeded into confocal dishes at a density of 1 × 10^5^ cells per well and incubated for 24 h. Afterward, the medium was replaced with fresh Dulbecco’s modified Eagle’s medium containing phosphate-buffered saline (PBS), PEG-MnMOF, PTX, or PEG-MnMOF@PTX [PEG-MnMOF (12.5 μg·ml^−1^) and PTX (2.63 μg·ml^−1^), respectively. After coculturing for another 24 h, the cells were rinsed and stained with DCFH-DA (10 μM in serum-free medium) under 37 °C for 30 min before CLSM, and the processing steps for FCM are similar.

The levels of intracellular GSH were evaluated using the GSH kit. CT26 cells were inoculated in 12-well plates and cultured at 1 × 10^5^ density for 24 h. The cells were then exposed to various treatments, i.e., PBS, PEG-MnMOF, PTX, and PEG-MnMOF@PTX. Later, the cells were collected, sonicated in an ice bath, and kept on ice for testing purposes. Then, 20 μl of the supernatant from the different treatment groups was mixed with 80 μl of dithionitrobenzoic acid in a 96-well plate. The absorbance of the final solution at 415 nm was measured by UV-vis spectroscopy.

For cell apoptosis assay, CT26 cells were seeded into 6-well plates and cultured for 24 h. Then, the cells were incubated with PBS, PEG-MnMOF, PTX, or PEG-MnMOF@PTX [PEG-MnMOF (12.5 μg·ml^−1^) and PTX (2.63 μg·ml^−1^)], respectively. Finally, cells were collected and stained with annexin V-fluorescein isothiocyanate (FITC)/propidium iodide (PI) for 15 min, followed by FCM analysis. The same treatment applies to confocal imaging.

### In vivo biodistribution and pharmacokinetic study

To study the in vivo biodistribution, the tumor-bearing mice were administrated with intravenous injection of PEG-MnMOF@PTX (200 μl, 1 mg·ml^−1^). Then, the major organs (heart, liver, spleen, lung, and kidney) and tumors were collected at different time points. The pharmacokinetic of PEG-MnMOF@PTX was evaluated in blood samples collected from BALB/c mice at 0.5, 1, 2, 4, 6, 8, 10, 12, and 24 h after intravenous injection of PEG-MnMOF@PTX. All samples were subjected to inductively coupled plasma-optical emission spectroscopy to obtain the Mn mass in each sample (percentage of injected dose per gram of tissue).

### Evaluation of ICD and DCs maturation

CT26 cells were seeded in confocal dish at a density of 1×10^5^ cells per dish and cultured for 24 h. Subsequently, the medium was replaced with fresh medium containing PBS, PEG-MnMOF, PTX, or PEG-MnMOF@PTX with various concentrations and cocultured for another 24 h. After that, the cells were stained with calreticulin (CRT) antibody to detect by CLSM. In addition, the supernatants were collected, and the released high mobility group box-1 protein (HMGB1) was detected using an enzyme-linked immunosorbent assay (ELISA) kit. The extracellular adenosine 5′-triphosphate (ATP) content was measured with an ATP Assay Kit (Beyotime, China) according to the manufacturer’s instructions.

For in vitro DCs maturation examination, the Transwell system was used for the coincubation of CT26 cells and bone marrow-derived DCs (BMDCs). In a typical setting, we placed CT26 cells in the upper chambers after different sample treatments, and BMDCs were seeded in the lower 12-well plate and incubated for a total of 24 h. Then, the BMDC was collected and stained with anti-CD11c APC anti-CD80 FITC, and anti-CD86 phycoerythrin (PE) for FCM analysis.

### Western blot analysis

First, CT26 cells were seeded in a 6-well plate at a density of 2 × 10^5^ overnight and treated with PBS, PEG-MnMOF, PTX, or PEG-MnMOF@PTX with various concentrations for 24 h, respectively. Afterward, the tumor cells were collected, and total protein concentrations were determined by a bicinchoninic acid protein assay kit. Then, the samples are dissolved in 1× SDS-polyacrylamide gel electrophoresis loading buffer and transferred onto a polyvinylidene fluoride membrane. Subsequently, the polyvinylidene fluoride membranes are labeled with primary antibodies, including STING [Cell Signaling Technology (CST)], phosphorylated STING (*p*-STING) (CST), TBK (CST), *p*-TBK1 (CST), cGAS (CST), and vinculin (CST). Then, corresponding secondary antibodies were introduced for incubation. Finally, imaging was performed using chemiluminescence detection reagents (KF001, Affinity), and the quantification was carried out with ImageJ.

### In vivo antitumor performance

Male BALB/c mice were injected with CT26 cells (1 × 10^6^) in the right back. The mice were randomly divided into 4 groups (*n* = 5). When the tumor volumes reached about 50 mm^3^, intravenous injection with different formulations was administrated on days 0, 3, and 7. These formulations include (a) PBS (control group), (b) PEG-MnMOF, (c) PTX, and (d) PEG-MnMOF@PTX. The dosages of PEG-MnMOF and PTX were 10 and 2 mg·kg^−1^ in related groups, respectively. The tumor size (*V*) was calculated as follows: *V* = width^2^ × length / 2 and measured every 2 d and photographed every 4 d. After 2 weeks, tumors tissues were weighed and collected. To observe the histopathological changes, we stained these slices with hematoxylin and eosin (H&E) staining, terminal deoxynucleotidyl transferase deoxyuridine triphosphate nick end labeling (TUNEL), and proliferating cell nuclear antigen (PCNA).

To better evaluate the therapeutic efficacy of PEG-MnMOF@PTX combined with αPD-L1 on CT26-tumor-bearing mice, we randomly divided the tumor-bearing mice into 4 groups (*n* = 5): (a) control, (b) αPD-L1, (c) PEG-MnMOF@PTX, and (d) PEG-MnMOF@PTX + αPD-L1. After the mice received different formulations treatments, the tumor volume and the body weight of each group were measured every 4 d. Mice were euthanized when the tumor volume reached 1,000 mm^3^. The mice were observed for a 40-d survival analysis.

### In vivo antitumor immunity assessment

To study in vivo immunity, tumor-bearing mice were randomly divided into 4 groups—(a) PBS (control group), (b) PEG-MnMOF, (c) PTX, and (d) PEG-MnMOF@PTX (*n* = 5)—and injected intravenously with the corresponding drugs. On the third day after drug administration, blood was collected from the mice, and subcutaneous tumors were removed. The excised tumor tissues were digested with collagenase IV, hyaluronidase, and deoxyribonuclease I at 37 °C for 30 min. Single-cell suspensions were then obtained by filtration through a 40-μm cell filter to study DC maturation. Single-cell suspensions were stained with anti-CD11c FITC, anti-CD86 PE, and anti-CD80 APC and then subjected to FCM analysis. Subsequently, ELISA kits were used to determine the concentrations of cytokines interleukin-6 (IL-6) and tumor necrosis factor-α (TNF-α) in serum. On the eighth day after intravenous administration, single-cell suspensions were stained by anti-CD11b PerCP-CY5.5, anti-F4/80 FITC, anti-CD86 PE, and anti-CD206 APC to observe the macrophage M2. Single-cell suspensions were stained with anti-Gr-1 FITC and anti-CD11b PerCP-CY5.5 to study the aggregation of MDSCs. The serum levels of IL-10 and IL-12 were also measured by ELISA assay. In addition, to demonstrate the expression of CTL and T_reg_, tumor-bearing mice were divided into 4 groups: (a) control, (b) αPD-L1, (c) PEG-MnMOF@PTX, and (d) PEG-MnMOF@PTX + αPD-L1; tumors from mice were removed; single-cell suspensions were prepared and stained with anti-CD3 PE, anti-CD4 FITC, anti-CD8 APC and anti-CD3 PE, anti-CD4 FITC, and anti-FOXP3 APC staining to study the infiltration of CTL and T_reg_ in mouse tumors; and the serum levels of type 1 IFN-γ was measured by ELISA.

### MRI property

MRI was conducted under a Siemens 3.0T MR with a special coil for small animal imaging (repetition time, 14.0; echo time, 2.8). First, we established MRI signal correction curves by scanning the T1-weighted signal intensity of several concentrations of PEG-MnMOF (12.5, 25, 50, 100, and 200 μg·ml^−1^) in aqueous solution. The imaging performance of PEG-MnMOF in mice was then further assessed by MR images of tumor-bearing mice 6 h after intravenous injection of PEG-MnMOF.

### Biosafety assessment

Mice were randomly divided into 4 groups (*n* = 5) and observed and evaluated at 0, 7, 14, and 30 d after intravenous injection of PEG-MnMOF (200 μl, 1 mg·ml^−1^). The major organs (heart, liver, spleen, lung, and kidney) were fixed after the last injection for H&E staining to assess toxicity. The hepatic and renal functions were assessed through blood tests.

### Statistical analysis

Data were expressed as means ± SD by one-way analysis of variance (ANOVA). *P* values less than 0.05 were considered statistically significance (*P < 0.05, **P < 0.01, and ***P < 0.001).

## Results

### Synthesis and characterization of PEG-MnMOF@PTX

**Fig. 2.**
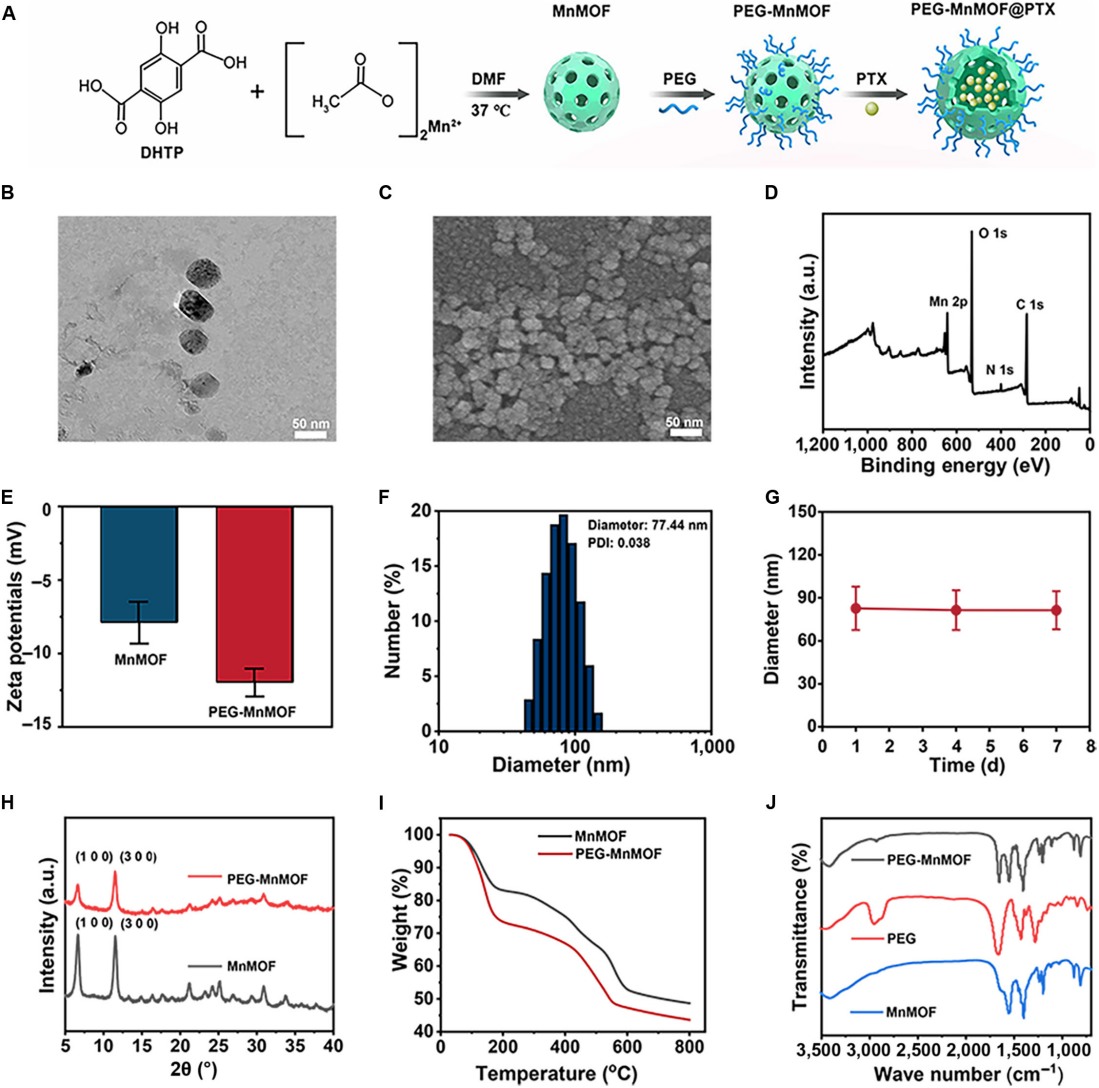
Preparation and characterization of PEG-MnMOF. (A) Schematic illustration of the process of preparation of PEG-MnMOF@PTX nanosystem. (B) Representative TEM image of PEG-MnMOF nanosystem. Scale bar, 50 nm. (C) Representative SEM image of PEG-MnMOF nanosystem. Scale bar, 50 nm. (D) Full-range survey XPS spectrum for PEG-MnMOF nanosystem. a.u., arbitrary units. (E) Zeta potential of MnMOF and PEG-MnMOF. (F) Size distribution of PEG-MnMOF as measured by zetasizer nano. (G) Average size of PEG-MnMOF in aqueous solution (on 1, 4, and 7 d). (H) XRD patterns of MnMOF and PEG-MnMOF. (I) TG analysis profiles of MnMOF and PEG-MnMOF. (J) Fourier transform infrared spectrometry patterns of MnMOF, PEG, and PEG-MnMOF.

We synthesized PEG-MnMOF@PTX, a nanosystem with TME modulation characteristics, using a self-assembly method outlined in Fig. 2A. The TEM image and SEM depicted in Fig. 2B and C and Fig. S1 revealed that PEG-MnMOF is similar to spherical but irregular. XPS further analyzed the elemental composition of PEG-MnMOF nanosystem (Fig. 2D), confirming the presence of Mn, O, C, and N elements. The PEG-MnMOF nanosystems were developed by linking MnMOF to PEG through phosphate groups, aiming to improve in vivo dispersion and prolong blood circulation [26]. The zeta potentials were −7.89 ± 1.43 mV for MnMOF and −11.97 ± 0.96 mV for PEG-MnMOF, evidencing successful modification of PEG (Fig. 2E) [21]. Its hydrodynamic diameter was measured to be 77.44 nm with a polymer dispersity index (PDI) of 0.038, as shown in Fig. 2F. During a 7-d period, we conducted continuous monitoring of the particle size of PEG-MnMOF in physiological saline. The results, as depicted in Fig. 2G, demonstrated that the particle size of PEG-MnMOF has no substantial change within 7 d, confirming the stability of the material under physiological conditions. The XRD assessment of the bulk structure of MnMOF showed characteristic peaks for both MnMOF and PEG-MnMOF at 2θ values of 6.6° and 11.4°, suggesting that the crystal structure of MnMOF remained unchanged after PEG modification (Fig. 2H). TG identified a notable distinction between the materials, revealing 4.9% of PEG in PEG-MnMOF (Fig. 2I). Fourier transform infrared spectrometry analysis further corroborated the successful transition from MnMOF to PEG-MnMOF (Fig. 2J). Taking into account the effect of specific surface area on enzymatic reaction, the specific surface area of MnMOF were evaluated by the classical N_2_ adsorption–desorption analysis. The results showed that the specific surface area of MnMOF was 90.3341 m^2^·g^−1^ (Fig. S2). The substantial specific surface area is advantageous for augmenting enzymatic reaction speeds and positioning this nanosystem as a promising material for drug delivery [27]. In addition, the nanosystem effectively loaded the chemotherapeutic drug PTX with an encapsulation efficiency of 21.9 ± 0.12% as measured by high-performance liquid chromatography.

### In vitro enzymatic activity and MRI capability of PEG-MnMOF@PTX

**Fig. 3.**
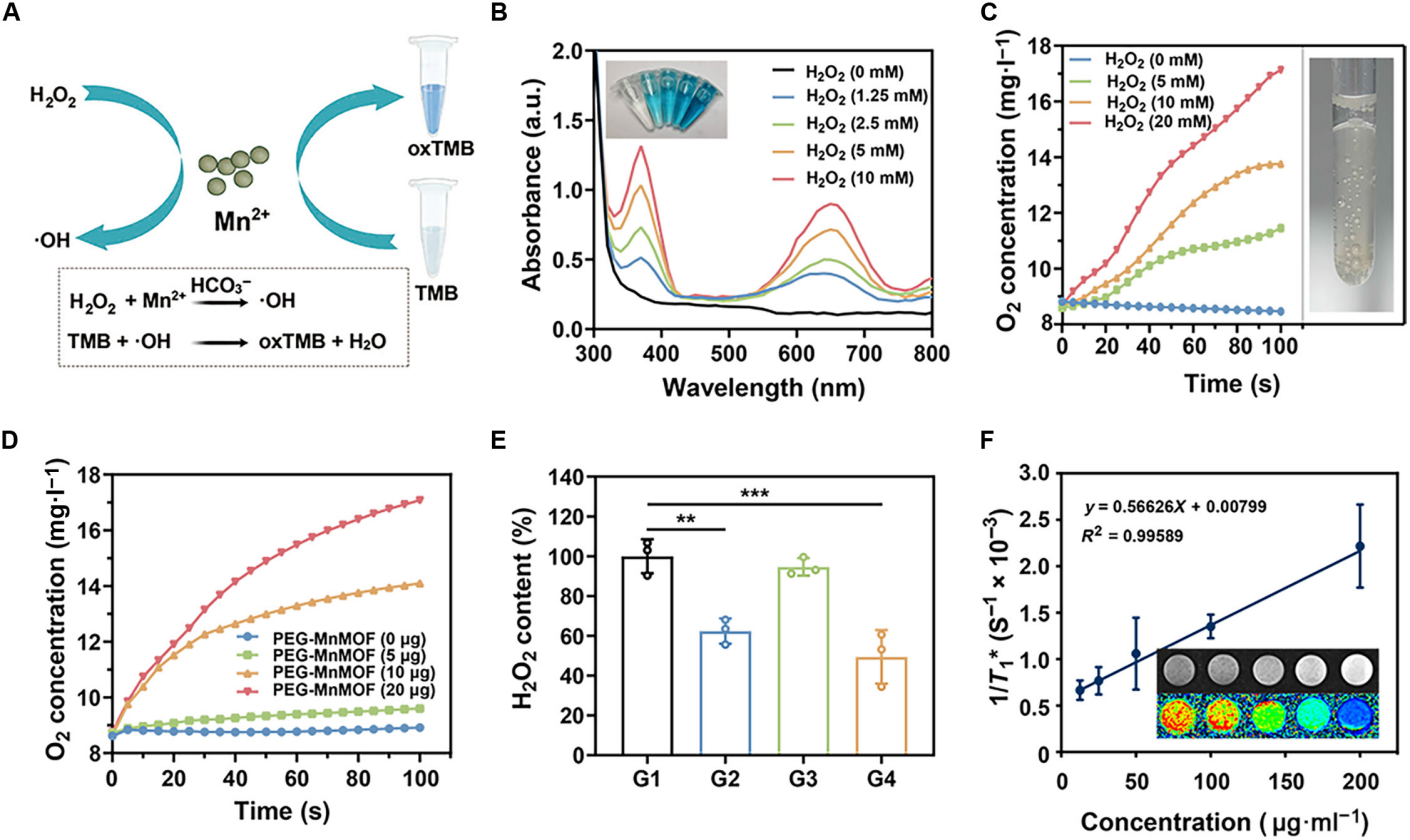
Multienzyme-like activity and GSH-depletion and in vitro MRI capabilities of PEG-MnMOF nanosystem. (A) Schematic illustration of the catalytic mechanism of PEG-MnMOF nanosystem. (B) Concentration-dependent •OH generation by PEG-MnMOF nanosystem, indicated by oxidation of TMB. (C and D) The oxygen production capacity of PEG-MnMOF with different concentrations of H_2_O_2_ and PEG-MnMOF, respectively. (E) Consumption of different groups of H_2_O_2_ upon the addition of different samples (G1, control; G2, PEG-MnMOF; G3, PTX; G4, PEG-MnMOF@PTX). (F) T1-weighted MR images of PEG-MnMOF@PTX and the reciprocal of T1 against the Mn concentration. Data are expressed as means ± SD (*n* = 3). Statistical significances were calculated via one-way ANOVA, **P < 0.01 and ***P < 0.001.

The PEG-MnMOF complex exhibited superior POD-like activity compared to other materials when Mn^2+^ was incorporated. It could generate •OH through the in situ decomposition of H_2_O_2_, facilitated by hydrocarbonate (HCO_3_^−^) as illustrated in Fig. 3A [28]. The •OH generated through the catalytic process can convert TMB into oxidized TMB (oxTMB) with a blue color, exhibiting peaks at 370 and 652 nm. The intensity of these characteristic peaks in Fig. 3B increased with the concentration of H_2_O_2_, and the blue solution exhibited a substantially greater depth. The findings suggest that PEG-MnMOF exhibits efficient generation of •OH and depletion of H_2_O_2_ through Mn^2+^-mediated Fenton-like reactions, highlighting the potential of PEG-MnMOF for CDT. Interestingly, PEG-MnMOF also demonstrated robust CAT-like activity. In the acidic milieu, we assessed the CAT activity of PEG-MnMOF and its capacity for oxygen production using a dissolved oxygen meter. The oxygen production capacity of PEG-MnMOF was analyzed in the presence of varying of H_2_O_2_, as depicted in Fig. 3C. Results showed that PEG-MnMOF exhibited stronger CAT-like activity and higher oxygen production capacity as the concentration of H_2_O_2_ increased. Many bubbles were observed in the paraffin-embedded liquid system (Fig. 3C, inset). Meanwhile, the catalytic efficiency of PEG-MnMOF clearly demonstrated a concentration-dependent relationship with the enzyme, thereby alleviating the hypoxia in TME (Fig. 3D). The enzyme activity assay was conducted at 37 °C to replicate the catalytic conditions in vivo. These findings were further validated through in vitro experiments examining H_2_O_2_ depletion (Fig. 3E). Considering that the potential of Mn^2+^ as a contrast agent for MRI [29], we investigated the imaging properties of the nanosystem. The longitudinal relaxation time of PEG-MnMOF@PTX in aqueous solution was investigated, as depicted in Fig. 3F. The r1 value was determined by performing a linear regression analysis on the reciprocal of T1 against the Mn concentration. The data revealed an enhanced T1-weighted signal as the concentration of PEG-MnMOF@PTX increased. The results demonstrate the excellent MRI capability of PEG-MnMOF@PTX, providing a valuable tool for monitoring the tumor accumulation of these nanosystems.

### Cellular phagocytosis, intracellular oxidative stress, and cytotoxicity of PEG-MnMOF@PTX nanosystem

**Fig. 4.**
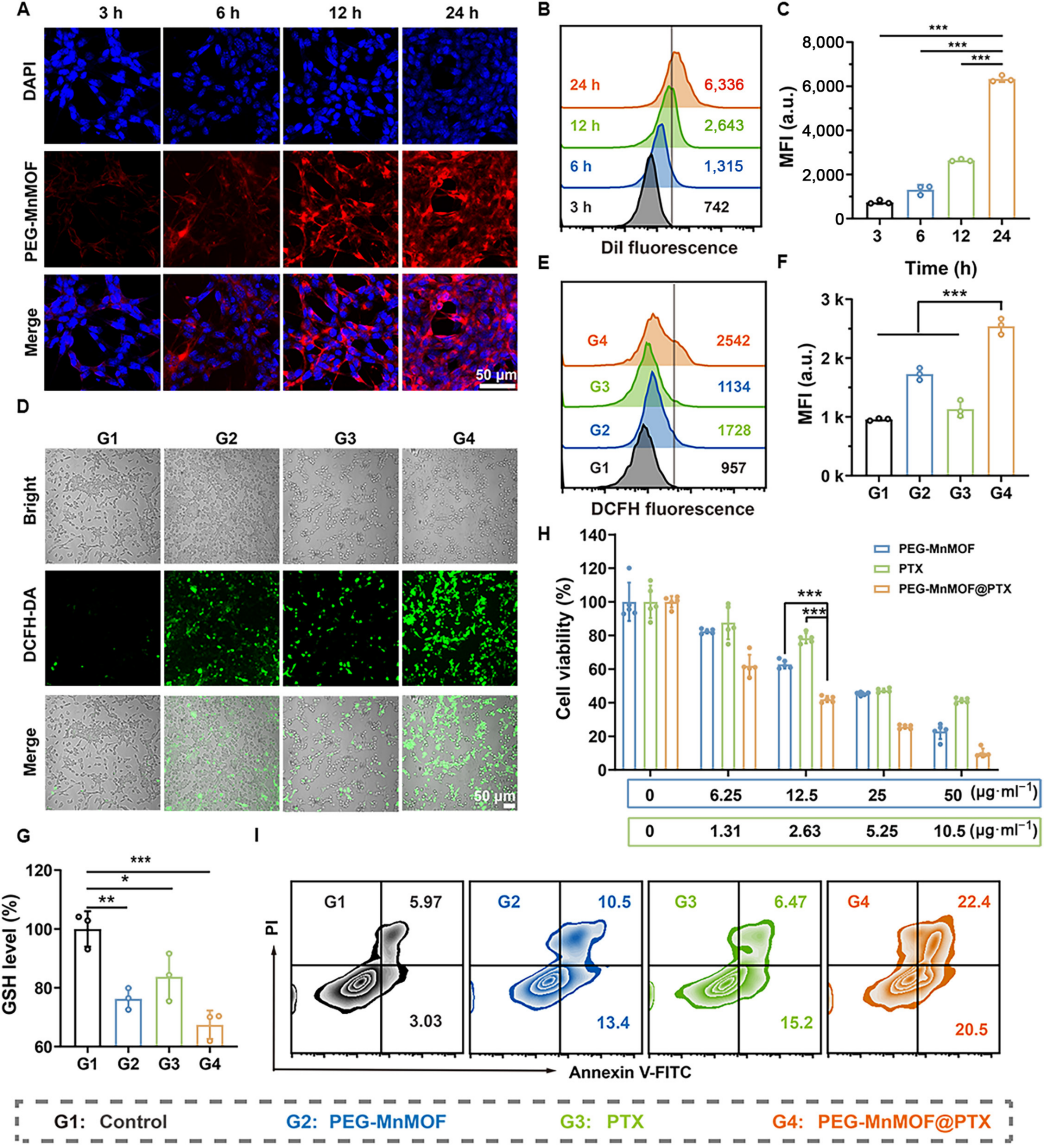
Intracellular uptake, oxidative stress, and cytotoxicity. (A) Intracellular uptake of PEG-MnMOF (labeled with DiI) detected by CLSM. Scale bar, 50 μm. DAPI, 4′,6-diamidino-2-phenylindole. (B and C) Intracellular uptake of PEG-MnMOF analyzed using FCM and the corresponding statistical analysis at different time points (*n* = 3). MFI, mean fluorescence intensity. (D) Intracellular ROS generation detected by CLSM. Scale bar, 50 μm. (E and F) Intracellular ROS generation assessed using FCM and the corresponding statistical analysis (*n* = 3). (G) Intracellular GSH levels after treatment with different simples (*n* = 3). (H) CCK-8 results after various treatments (*n* = 5). (I) Cell apoptosis detected using FCM after different treatments (stained with annexin V-FITC/PI apoptosis kit). Data are expressed as means ± SD. Statistical significances were calculated via one-way ANOVA, *P < 0.05, **P < 0.01, and ***P < 0.001.

The internalization of PEG-MnMOF by tumor cells was assessed by treating CT26 with DiI-labeled PEG-MnMOF. The fluorescence-labeled PEG-MnMOF was incubated with CT26 for different durations (3, 6, 12, and 24 h), and the intracellular fluorescence could be visualized using a CLSM. After 24 h of coincubation, cells exhibited robust red fluorescence, suggesting efficient internalization of PEG-MnMOF into CT26 cells (Fig. 4A). This observation was also supported by the FCM data (Fig. 4B and C).

After successfully demonstrating the efficient phagocytosis of PEG-MnMOF, we proceeded to investigate the mechanism by which PEG-MnMOF@PTX induces cell death, specifically focusing on its capacity to generate cytotoxic ROS and the intracellular GSH depletion. First, intracellular ROS levels were assessed using the ROS-probe DCFH-DA, which undergoes a reaction with 2,7-dichlorofluorescein diacetate and emits fluorescence in the green spectrum. The level of ROS production was observed by CLSM following various treatments. The green fluorescence exhibited the highest intensity in PEG-MnMOF@PTX group, as depicted in Fig. 4D, indicating a substantial generation of highly toxic ROS potentially originating from both PEG-MnMOF and PTX. The FCM data reinforced these findings (Fig. 4E and F). The pivotal role of GSH in cellular antioxidant systems prompted us to use a GSH assay kit for the evaluation of PEG-MnMOF@PTX’s capacity to deplete GSH. Substantial GSH reduction was observed after various treatments, with PEG-MnMOF@PTX showing the most pronounced effect (Fig. 4G). The aforementioned observation implies that the combination of PEG-MnMOF and PTX induces an elevation in ROS generation, disrupts cellular antioxidant equilibrium, and augments ROS sensitivity.

After investigating the impact of PEG-MnMOF@PTX on intracellular oxidative stress, we subsequently evaluated its cytotoxicity through CCK-8 assay, protein-linked annexin V-FITC/PI staining, and a live–dead staining kit. First, we assessed the cytotoxicity of the compound on CT26 cells using the standard CCK-8 assay. Notably, PEG-MnMOF@PTX exhibited superior cytotoxicity compared to either PEG-MnMOF or PTX alone at equimolar concentrations over a 24-h time frame (Fig. 4H), indicating the synergistic therapeutic effects of PEG-MnMOF and PTX on cells in vitro. As shown in Fig. 4I and Fig. S3A, notably, PEG-MnMOF@PTX exhibited substantially higher apoptosis rate (43.1%) than PEG-MnMOF alone and PTX (29.3% and 22.2%, respectively). The live–dead assay also reflected similar results, with pronounced red fluorescence in the PEG-MnMOF@PTX group (Fig. S3B). The findings suggest that the release of Mn^2+^ in acidic environment drives the conversion of endogenous H_2_O_2_ to the toxic •OH. When combined with PTX, it enhances intracellular ROS levels, resulting in irreversible cell death. Thus, the efficacy of the drug-loaded nanosystem in tumor eradication is well substantiated.

### In vitro stimulation and activation of antitumor immune responses

**Fig. 5.**
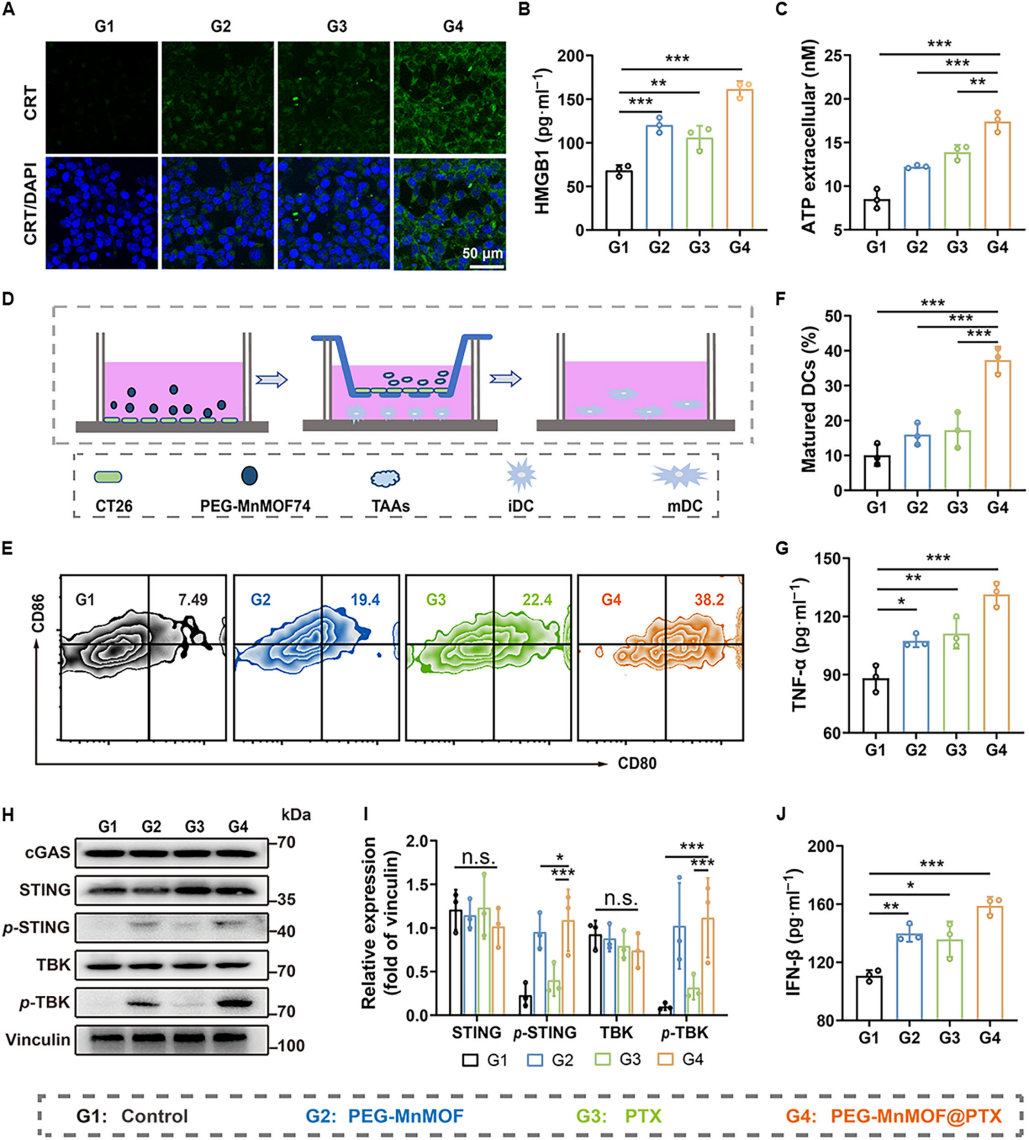
In vitro stimulation and activation of the antitumor immune response. (A) CLSM images of CRT exposure after various treatments. Scale bar, 50 μm. (B) Cytokine levels of HMGB1 in the supernatant medium of CT26 cancer cells after various treatments (*n* = 3). (C) ATP in CT26 suspensions after various treatments detected by the ATP assay kit (*n* = 3). (D) Schematic illustration of the coculture system. mDC, mature DC; iDC, immature DC. (E and F) Representative FCM results of matured DCs (CD11c^+^ CD80^+^ CD86^+^) after different treatments and the corresponding quantitative analysis of DC maturation (*n* = 3). (G) Cytokine levels of TNF-α in matured DCs suspensions (*n* = 3). (H and I) Western blot analysis of the STING-pathway-relative protein expression after various treatments and the corresponding statistical analyses (*n* = 3). (J) Secretion levels of IFN-γ in CT26 suspensions. Data are expressed as means ± SD. Statistical significances were calculated via one-way ANOVA, *P < 0.05, **P < 0.01, and ***P < 0.001. n.s., no significance.

The potential immunostimulatory effects of this combinational treatment were subsequently assessed by evaluating the release ability of various DAMPs (CRT, HMGB1, and ATP). The involvement of CRT signals in antigen presentation is crucial for the initiation of tumor-associated immune responses [30]. CT26 cells were subjected to various treatments, followed by staining with FITC-labeled CRT antibody, and subsequently observed using CLSM. The results demonstrated that both the PEG-MnMOF and PTX groups exhibited relatively weak fluorescence signals, whereas a robust green fluorescence was observed in the PEG-MnMOF@PTX group (Fig. 5A). A commercially available ELISA kit was utilized to quantify the extent of HMGB1, a distinct DAMP [31]. The levels of HMGB1 released from tumor cells after PEG-MnMOF, PTX, and PEG-MnMOF@PTX treatments were 1.76, 1.55, and 2.37 times higher than that in the control group, respectively, as illustrated in Fig. 5B. The release of ATP by dying cells also promotes the phagocytosis of APCs and augments specific antitumor effects [32,33]. We used an ATP assay kit to quantify extracellular ATP levels and observed that the combination of PEG-MnMOF and PTX treatment group exhibited the most efficient (Fig. 5C). Given that CRT, HMGB1, and ATP are established biomarkers of ICD, our findings suggest a potential synergistic effect of PEG-MnMOF and PTX in promoting optimal ICD induction in tumor cells.

In addition, Mn^2+^ has been reported to facilitate DC maturation through the STING pathway, thereby triggering robust antitumor responses [34]. Given the ICD induction capacity of PEG-MnMOF@PTX, its potential for in vitro activation of antitumor immunity was determined next. The maturation potential of DCs, which play a crucial role in immune response [35], was evaluated using a Transwell system in the presence of various treated tumor cells. We seeded treated CT26 cells in the upper chambers and immature DCs in the lower chambers, and the cells were coincubated for 24 h (Fig. 5D). The cells in the lower chamber were harvested and stained with anti-CD11c APC anti-CD80 FITC, and anti-CD86 PE. FCM analysis was performed, and cell supernatants were collected to measure proinflammatory cytokine secretion using an ELISA kit. PEG-MnMOF-PTX demonstrated superior efficacy in promoting DC maturation, as evidenced by the results shown in Fig. 5E and F, compared either PEG-MnMOF or PTX alone. Meanwhile, the PEG-MnMOF@PTX group exhibited substantially higher expression levels of IL-6 and TNF-α, which were secreted by DCs during the maturation process (Fig. 5G and Fig. S4).

It was also observed that the PEG-MnMOF@PTX group efficiently induced the secretion of IFN-β [36], a characteristic cytokine activated by the STING pathway (Fig. 5J). Furthermore, to demonstrate the activation of the STING pathway by Mn^2+^, we conducted Western blot analysis to assess the phosphorylation levels of STING proteins and their downstream TBK proteins. The expression of *p*-STING and *p*-TBK was prominently observed in the Mn-based group, whereas it was comparatively weaker in the free PTX-treated group (Fig. 5H and I). In summary, the PEG-MnMOF@PTX nanosystem exhibits a heightened ICD induction compared to individual agents, and the presence of Mn^2+^ enhances the activation of STING signaling pathway associated with ICD induction.

### The biodistribution/pharmacokinetic of PEG-MnMOF@PTX

We then evaluated the detailed in vivo biodistribution and pharmacokinetic behaviors of PEG-MnMOF@PTX by inductively coupled plasma-optical emission spectroscopy. As observed for the biodistribution of most of the nanosystem in vivo, the nanosystem tends to accumulate in the liver and spleen rather than in the tumor at 2 h after injection, possibly due to entrapment by the reticuloendothelial system (Fig. S5A). However, with increasing time, the accumulation in the liver and spleen decreased substantially, while the tumor reduction was less than that in the liver and spleen. Simultaneously, the pharmacokinetics of the [Mn] content in blood shows a classical 2-compartment model. It was then determined that the first and second half-lives of PEG-MnMOF@PTX were calculated to be 0.46 ± 0.20 h and 3.73 ± 0.85 h (Fig. S5B). The above results demonstrated that PEG-MnMOF@PTX possessed the capacity of efficient tumor accumulation and rapid clearance from the blood and the major organs. These excellent biodistribution and pharmacokinetic behaviors indicate that it can be safely used as a nanotherapeutic reagent for in vivo therapy.

### In vivo antitumor therapy with PEG-MnMOF@PTX

For in vivo applications of biomaterials, biosafety is paramount. The in vivo biosafety of PEG-MnMOF@PTX was initially assessed. After intravenous administration into healthy male BALB/c mice, blood and major organs were collected from each group at different time points (0, 7, 14, and 30 d) for subsequent analysis. The blood routine and blood biochemical indices (liver function, kidney function, and heart function) of mice treated with PEG-MnMOF@PTX showed no significant difference compared to untreated mice, as depicted in Fig. S6A. In addition, the H&E staining results of major organs (heart, liver, spleen, lung, and kidney) showed no substantial pathological changes (Fig. S6B). These findings indicate that PEG-MnMOF@PTX exhibits a favorable biosafety profile. The CT26-tumor-bearing mice were intravenously injected with PEG-MnMOF@PTX, followed by MRI. The T1-MR signals exhibited a specifically in the tumor region, indicating a substantial accumulation of PEG-MnMOF@PTX within the tumor (Fig. S7A and B).

**Fig. 6.**
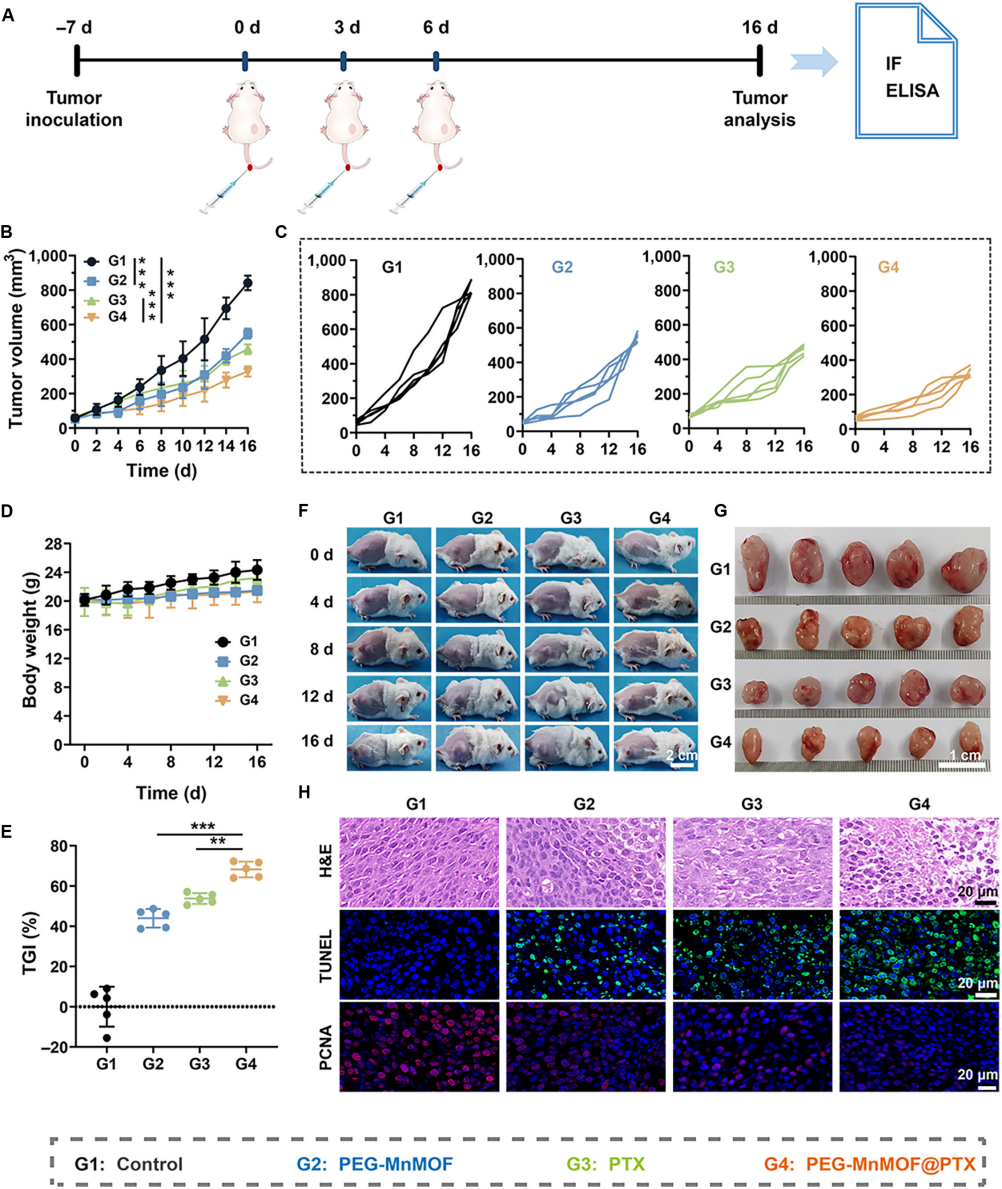
Antitumor immune response in vivo. (A) Schematic illustration of the experimental schedule on tumor-bearing mice. IF, immunofluorescence. (B and C) Tumor volume changes of 4 groups after various treatments (*n* = 5). (D) Body weight changes of tumor-bearing mice during treatment (*n* = 5). (E) TGI of tumors (*n* = 5). (F) Photograph of tumor-bearing mice during treatment. Scale bar, 2 cm. (G) The image of the dissected tumors on day 16. Scale bar, 1 cm. (H) H&E, TUNEL, and PANC staining of tumor sections after different treatments. Scale bars, 20 μm. Data are expressed as means ± SD. Statistical significances were calculated via one-way ANOVA, **P < 0.01 and ***P < 0.001.

Afterward, to assess the antitumor efficacy of the nanosystem, we established mouse CT26 tumor models. When the tumor volume reached 50 mm^3^, CT26-tumor-bearing mice were randomly divided into 4 groups (*n* = 5), including (a) PBS, (b) PEG-MnMOF, (c) PTX, and (d) PEG-MnMOF@PTX. The treatment schedule is outlined in Fig. 6A, wherein the body weight and tumor volume of mice were recorded every 2 d and the tumor sites of mice were photographed every 4 d to record tumor changes. The growth curves of the tumors showed that PEG-MnMOF@PTX had the most substantial antitumor effect compared to the control (Fig. 6B, C, and F). The mice exhibited no substantial alterations in body size or weight following injection of PEG-MnMOF@PTX, indicating the safety of the administered dose for mice (Fig. 6D). Then, all mice were euthanized at the end of the treatment on day 16. Tumors were dissected, photographed, and weighed to calculate tumor growth inhibition (TGI). As shown in Fig. 6E and G, both the PEG-MnMOF and PTX groups exhibited moderate tumor inhibition effects (44.0% and 53.8%, respectively), whereas the PEG-MnMOF@PTX group demonstrated the most substantial inhibition with a TGI rate as high as 68.2%. These findings suggest that Mn^2+^ synergizes with PTX-induced DNA damage to activate the cGAS–STING pathway in enhancing antitumor immunity.

Furthermore, tumor cell necrosis and apoptosis were observed by H&E staining and TUNEL immunofluorescence staining, and tumor cell proliferation was assessed using PCNA staining. The H&E staining results revealed a substantially higher degree of fragmentation and nucleolysis in tumor cells from mice treated with PEG-MnMOF@PTX compared to the other 3 groups. TUNEL staining showed that the green fluorescence of the PEG-MnMOF@PTX-treated group was substantially stronger than that of the PEG-MnMOF and PTX groups, indicating a pronounced induction of apoptosis by PEG-MnMOF@PTX. The PCNA analysis revealed that the PEG-MnMOF@PTX group exhibited the most potent inhibition of tumor cell proliferation, highlighting the combination on combating tumors (Fig. 6H).

### PEG-MnMOF@PTX enhances immune response in vivo

**Fig. 7.**
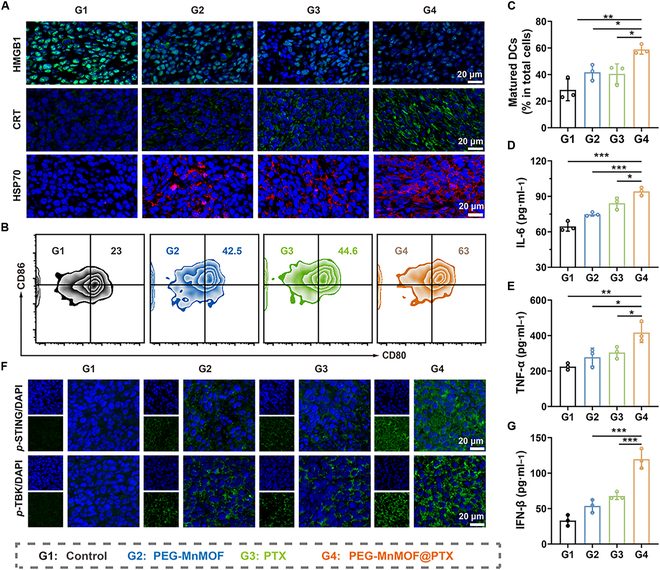
In vivo ICD, DC maturation, and activation of cGAS–STING pathway. (A) Immunofluorescence images of tumor slices stained by CRT, HMGB1, and heat shock protein 70 (HSP70). Scale bars, 20 μm. (B and C) Representative images of mature DCs (CD11c^+^ CD86^+^ CD80^+^) analyzed by FCM and the corresponding quantitative analysis after treatment with different samples (*n* = 3). (D and E) Serum levels of TNF-α and IL-6 in CT26-tumor-bearing mice after different treatments (*n* = 3). (F) Immunofluorescence images of tumor slices stained by *p*-STING and *p*-TBK. Scale bars, 20 μm. (G) Serum levels of IFN-β in CT26-tumor-bearing mice after different treatments (*n* = 3). Data are expressed as means ± SD. Statistical significances were calculated via one-way ANOVA, *P < 0.05, **P < 0.01, and ***P < 0.001.

On the basis of the ability to stimulate and activate antitumor immune responses in vitro, the in vivo potential of PEG-MnMOF@PTX to induce ICD was investigated. Following various treatments, we conducted immunofluorescence staining to analyze the expression of HMGB1 and CRT in tumor tissues. As shown in Fig. 7A, in the PEG-MnMOF@PTX group, the least expression of HMGB1 and the strongest fluorescence of CRT was observed, suggesting substantial HMGB1 release and elevated expression of CRT. DCs are capable of identifying these typical DAMPs, thereby enhancing their antigen-presenting function and subsequently facilitating T cell infiltration [37]. To assess the in vivo antitumor immune responses, we performed tumor dissection following various treatments and prepared single-cell suspensions. Subsequently, the in vivo maturation rate of DCs (CD11c^+^ CD80^+^ CD86^+^) was analyzed using FCM. As shown in Fig. 7B, the PEG-MnMOF@PTX group exhibited the highest DC maturation rate of 58.9%, which was 2.1 times higher than that of the control group (Fig. 7C). Furthermore, we assessed the serum levels of proinflammatory cytokines, including IL-6 and TNF-α. Consistent with the enhanced DC maturation, a substantial surge in these cytokines was also observed (Fig. 7D and E).

In addition, we discovered that Mn^2+^ has the potential to activate the cGAS–STING signaling pathway, thereby augmenting the cross-presentation of DCs through IFN-β production and subsequently activating tumor-specific CD8^+^ T cells. To track the effect of PEG-MnMOF@PTX on STING pathway, we performed *p*-STING and *p*-TBK1 immunofluorescence staining on tumor tissues. Substantially stronger green fluorescence of *p*-STING and *p*-TBK1 was observed in the PEG-MnMOF@PTX group (Fig. 7F). We have also discovered that IFN-β secretion was 3.6 times higher than the control (Fig. 7G). These findings reinforced the PEG-MnMOF@PTX-enhanced antitumor immunity via ICD stimulation and STING pathway activation.

**Fig. 8.**
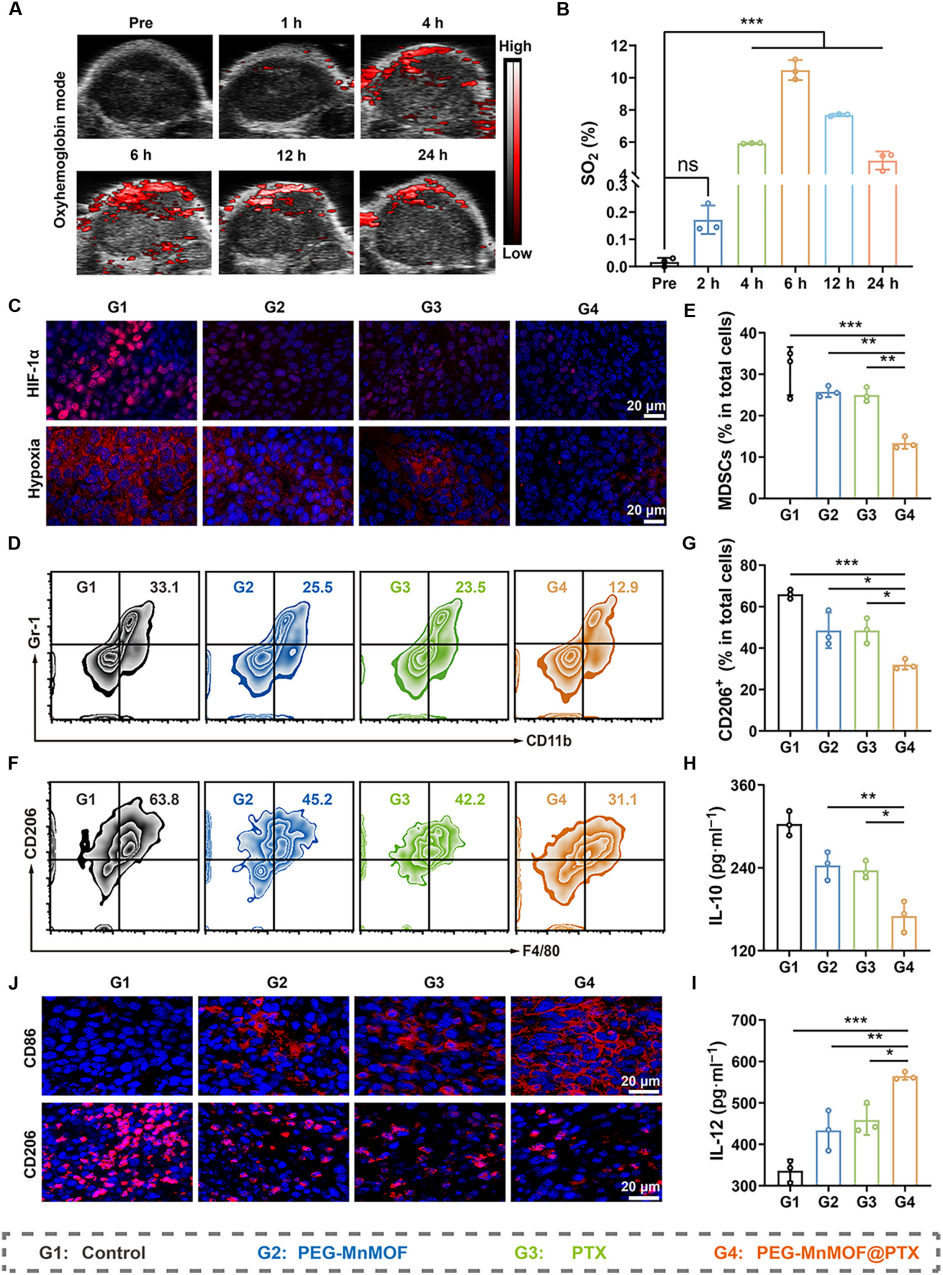
In vivo immunosuppression alleviation. (A and B) Representative photoacoustic images of CT26 tumor treated with PEG-MnMOF at different time points in oxyhemoglobin mode and the corresponding quantitative analysis (*n* = 3). (C) Immunofluorescence images of tumor slices stained by HIF-1α and pimonidazole (Pimo). Scale bars, 20 μm. (D and E) Representative images of MDSC (CD11b^+^ Gr-1^+^) analyzed by FCM and the corresponding quantitative analysis after various treatments (*n* = 3). (F and G) Representative images of M2-TAMs (CD11b^+^ F4/80^+^ CD206^+^) analyzed by FCM and the corresponding quantitative analysis after various treatments (*n* = 3). (H and I) Serum levels of IL-10 and IL-12 in CT26-tumor-bearing mice after various treatments (*n* = 3). (J) Immunofluorescence images of tumor slices stained by CD206 and CD86. Scale bars, 20 μm. Data are expressed as means ± SD. Statistical significances were calculated via one-way ANOVA, *P < 0.05, **P < 0.01, and ***P < 0.001.

Although elevated levels of ICD can strengthen T-cell-mediated antitumor immune responses, the immunosuppressive TME, often characterized by hypoxia, substantially compromises antitumor activity. Given the CAT-like activity of PEG-MnMOF, we evaluated its ability to alleviate the hypoxia in vivo. To visualize the oxygen production, we used a photoacoustic imaging system for the detection of oxygen saturation (SO_2_) at the tumor site (Fig. 8A) [38]. Interestingly, after intravenous administration of PEG-MnMOF@PTX, the oxygen saturation signal (red) within the tumor exhibited a substantial increase and reached its peak at 6 h after injection (Fig. 8B). The impact of PEG-MnMOF@PTX on tumor vessels was demonstrated by staining blood vessels with anti-CD31 antibody (red) and anti-α-smooth muscle actin (αSMA) antibody (green). The αSMA is essential for tumor angiogenesis and is considered a marker of myofibroblasts [39]. The treatment with different drugs resulted in a substantial increase in green and red fluorescence compared to the control group (Fig. S8), which means that the accumulation of Mn^2+^ in tumors triggers the decomposition of H_2_O_2_ into O_2_, leading to a substantial reduction in intratumoral hypoxia. In addition, tissue immunofluorescence staining revealed a reduction in hypoxic area and a decrease in HIF-1α expression following various treatments (Fig. 8C). These results indicate the ability of PTX to normalize the tumor vasculature, as well as the ability of the PEG-MnMOF@PTX nanosystem localized within the tumor to catalytically decompose H_2_O_2_ into O_2_.

The impact of PEG-MnMOF@PTX on the immunosuppressive TME was comprehensively assessed by analyzing M2-TAMs (CD11b^+^ F4/80^+^ CD206^+^) and infiltration of MDSC (CD11b^+^ Gr-1^+^) in the tumor cells using FCM. The levels of M2-TAMs and MDSC were substantially reduced in the PEG-MnMOF, PTX, and PEG-MnMOF@PTX groups compared to the control group, as depicted in Fig. 8D and F. Notably, the PEG-MnMOF@PTX group exhibited the most pronounced reduction (Fig. 8E and G). As shown in Fig. 8H and I, the ELISA assay demonstrated that the PEG-MnMOF@PTX group substantially down-regulated IL-10 expression and up-regulated IL-12 secretion, indicating a synergistic effect of PEG-MnMOF and PTX on M2-TAM polarization and reduced MDSC infiltration. These findings were further supported by immunofluorescence staining results (Fig. 8J).

### Combining PEG-MnMOF@PTX with αPD-L1 for enhanced antitumor immunotherapy

**Fig. 9.**
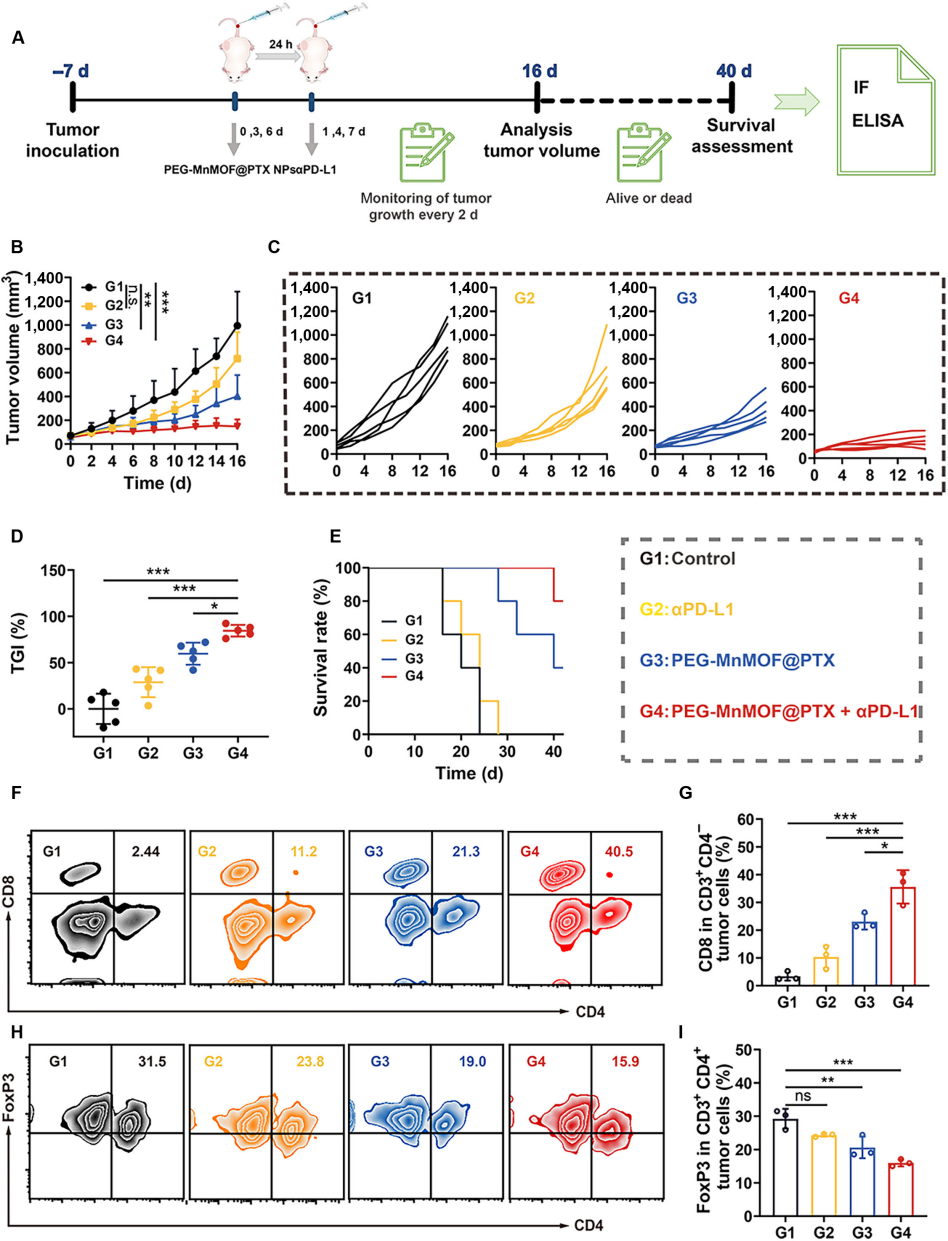
Combination of PEG-MnMOF@PTX with ICB therapy in vivo. (A) Schematic illustration of the experimental schedule on tumor-bearing mice. (B and C) Tumor volume changes of the 4 groups after various treatments (*n* = 5). (D) TGI of tumors (*n* = 5). (E) Survival curves of the tumor-bear mice after various treatments (*n* = 5). (F and G) Representative FCM results of CD8^+^ T (CD3^+^ CD4^−^ CD8^+^) analyzed by FCM and the corresponding quantitative analysis after various treatments (*n* = 3). (H and I) Representative FCM results of T_regs_ (CD3^+^ CD4^+^ FoxP3^+^) analyzed by FCM and the corresponding quantitative analysis after various treatments (*n* = 3). Data are expressed as means ± SD. Statistical significances were calculated via one-way ANOVA, *P < 0.05, **P < 0.01, and ***P < 0.001.

The remarkable capacity to stimulate antitumor immune responses and reprogram the immunosuppressive microenvironment position PEG-MnMOF@PTX as a promising immunomodulator. The inhibitory effect of the combination of αPD-L1 with our compound was subsequently assessed. We established CT26-tumor-bearing mice and randomly divided the mice into 4 groups (*n* = 5), including PBS, αPD-L1, PEG-MnMOF@PTX, and PEG-MnMOF@PTX + αPD-L1. The treatment schedule is depicted in Fig. 9A. The PEG-MnMOF@PTX and PEG-MnMOF@PTX + αPD-L1 groups demonstrated enhanced tumor suppression and prolonged survival in mice, as depicted in Fig. 9B to D. While PEG-MnMOF@PTX monotherapy had a 40-d survival rate of 40%, the combined therapy saw this rate jump to 80%, equivalent to that of the αPD-L1-only group and surpassing the control (Fig. 9E). This superior ability to inhibit tumor growth can be attributed to the activation of tumor-specific immune responses and the relief of immunosuppression mediated by PTX and Mn^2+^. The aforementioned modification facilitates the activation, proliferation, and infiltration of T cells into tumors, thereby enhancing the efficacy of ICB treatment.

To decipher the intrinsic mechanism of the high efficiency of PEG-MnMOF@PTX + αPD-L1, the investigation was conducted to examine its influence on the delivery of antigens. Considering that mature DC can deliver peptide–major histocompatibility complex to naïve T lymphocytes, promote CD8^+^ T cells infiltration, and antagonize T_reg_ production, as well as the relief of the immunosuppression, we evaluated the levels of CD8^+^ T cells and T_regs_. After various treatments, the proportion of CTL CD8 (CD3^+^ CD4^−^ CD8^+^) and T_regs_ (CD3^+^ CD4^+^ FoxP3^+^) was quantified using FCM (Fig. 9F to I). We observed a substantial 10.4-fold increase in PEG-MnMOF@PTX + αPD-L1 treatment group compared to the control group, while there was a notable decrease in the proportion of T_regs_ (16.0% versus 29.2% in control). The immunofluorescence staining exhibited a comparable pattern (Fig. S9). The antitumor immune-related IFN-γ level was found to be consistent with the results obtained from FCM (Fig. S10).

## Discussion

In recent years, ICB has shown great promise as an effective cancer treatment. However, the therapeutic efficacy of ICB is severely hampered by insufficient T cell infiltration and tumor immunosuppressive microenvironment, allowing a small group of patients to profit from it [40]. Thus, it is vital to transform cold tumors with poor immunogenicity into hot tumor with high immunogenicity. In this work, we designed and developed PEG-MnMOF@PTX to enhance the immunotherapeutic efficiency of ICB. The antitumor mechanism of PEG-MnMOF@PTX in combination with αPD-L1 can be summarized as follows, essentially. (a) The activity of POD-like enzymes, in conjunction with PTX, triggers the production of cytotoxic ROS, induces ICD, and escalates the tumor-specific immune response. (b) The hypoxia TME can be alleviated by the decomposition of intratumoral H_2_O_2_ into O_2_ via CAT-like enzymes activity and the normalization of tumor blood vessels, thereby reducing the frequency of immunosuppressive cells such as M2-TAMs, MDSCs, and T_regs_. (c) The combination of Mn^2+^ and PTX synergistically triggers the activation of the cGAS–STING pathway, which promotes antigen presentation, and the activation of tumor-specific CD8^+^ T cells. (d) The conversion from irresponsiveness to immunogenic susceptibility in tumors enhances CD8^+^ T infiltration and augments the antitumor efficacy, thereby boosting the response to ICB therapy [41]. Furthermore, Mn^2+^ can be utilized for MRI to detect the accumulation of PEG-MnMOF tumor sites. Therefore, the utilization of PEG-MnMOF@PTX nanosystem in conjunction with αPD-L1 presents an innovative paradigm for clinical tumor treatments.

Presently, a variety of therapies are available to enhance ICB by improving tumor immunogenicity, which can be broadly classified into 3 categories: chemotherapy, thermotherapy, and ROS-related therapies [42]. Traditional chemotherapy has serious systemic side effects, while PEG-MnMOF@PTX, as a TME-responsive material, can selectively deliver chemotherapeutic drugs to the tumor to reduce potential side effects [43]. Thermotherapy (e.g., photothermal therapy, high-intensity-focused ultrasound ablation, and radiofrequency ablation) tends to damage nearby normal tissues, but decreasing the ablation temperature or time to reduce undesired damage can easily prone to incomplete ablation of tumor and further promote tumor recurrence. The CDT, relies on high levels of H_2_O_2_ in TME, is a safe treatment modality with a high therapeutic specificity. Most of ROS-mediated therapies (e.g., sonodynamic therapy, photodynamic therapy, and radiotherapy) are oxygen dependent, and the hypoxic microenvironment of tumors limits ROS generation, whereas CDT is oxygen-independent therapeutic modality [44]. In addition, unlike conventional drug delivery system, the PEG-MnMOF@PTX not only acts as a drug delivery system to transport the chemotherapeutic drug PTX to the tumor site but also has the effect of enhancing immunogenicity and reprogramming of tumor immunosuppression. The nanosystem effectively overcame hypoxia-induced immune resistance, promoted macrophage polarization, and reduced the proportion of immunosuppressive M2-TAMs, T_regs_, and MDSCs. Furthermore, as a potent agonist that can activate the STING pathway independently of dsDNA, Mn^2+^ could synergize with PTX-mediated DNA damage to jointly enhance the cGAS–STING pathway and stimulate DCs maturation. The above experimental results show that the nanosystem, such as PEG-MnMOF@PTX, provides an effective therapeutic scheme for the clinical treatment of tumors.

## Conclusion

In summary, the PEG-MnMOF@PTX composite with TME-modulating properties was successfully synthesized via self-assembly to enhance the sensitivity of ICB immunotherapy by amplifying STING activation and alleviating immunosuppression. PEG-MnMOF@PTX exhibits intrinsic POD and CAT-like activities, resulting in the generation of cytotoxic •OH and oxygen molecules. The induction of oxidative-damage-mediated ICD and the amelioration of intratumoral hypoxia respectively enhance the immunogenicity and reprogram the tumor immunosuppression. The frequencies of immunosuppressive cells, including M2-TAMs, MDSCs, and T_regs_ were substantially diminished, while the infiltration of T cells was augmented. Moreover, the STING agonists, PTX and Mn^2+^, act synergistically to activate the cGAS–STING pathway, thereby facilitating the maturation of DCs. Notably, PEG-MnMOF@PTX demonstrated potent induction of proinflammatory factors TNF-α and IL-6 secretion, along with TGI evidenced by a tumor suppression of up to 84.4% and a substantial improvement in survival. The findings suggest that PEG-MnMOF@PTX hybrid nanosystems possess the potential to enhance the efficacy of tumor immunotherapy and offer novel prospects for future clinical antitumor therapy.

### Ethics Approval and Consent to Participate

All animal experiments complied with the relevant ethical standards and all procedures were approved by the Animal Experimentation Ethics Committee of The First Affiliated Hospital of Chongqing Medical University.

## Data Availability

The datasets used and/or analyzed during the current study are available from the corresponding author on reasonable request.
