# Supplementary material for: Amplifying STING Activation and Alleviating Immunosuppression through a Mn2+-Based Metal-Organic Framework Nanosystem for Synergistic Cancer Therapy
Source: Biomater Res. 2024 May 2;28:0028. doi: 10.34133/bmr.0028 (PMC11075671; doi:10.34133/bmr.0028)
Supplement: Supplementary 1 — Figs. S1 to S10 [file bmr.0028.f1.docx]

***Supplemental Materials for***

**Amplifying STING Activation and Alleviating Immunosuppression through a Mn^2+^-based Metal-Organic Framework Nanosystem for Synergistic Cancer Therapy**

Mingxiao Fang^1†^, Jun Zheng^1†^, Jingxue Wang^1^, Chenpeng Zheng^3^, Xiaojing Leng^1^, E Wen^1*^, Pan Li^1^, Haitao Ran^1^, Liang Zhang^1, 2*^, Zhigang Wang^1*^

^1^State Key Laboratory of Ultrasound in Medicine and Engineering, Institute of Ultrasound Imaging, The Second Affiliated Hospital, Chongqing Medical University, Chongqing 400010, PR China

^2^Ultrasound Department, The First Affiliated Hospital of Chongqing Medical University, Chongqing 400042, PR China

^3^Chongqing Emergency Medical Center, Chongqing University Central Hospital, Chongqing, 400014, P.R. China.

Mingxiao Fang and Jun Zheng contributed equally to this work.

***Corresponding authors:** [we_we11@163.com](mailto:we_we11@163.com); [zhangliang338@cqmu.edu.cn](mailto:zhangliang338@cqmu.edu.cn); [wangzhigang@cqmu.edu.cn](mailto:wangzhigang@cqmu.edu.cn)


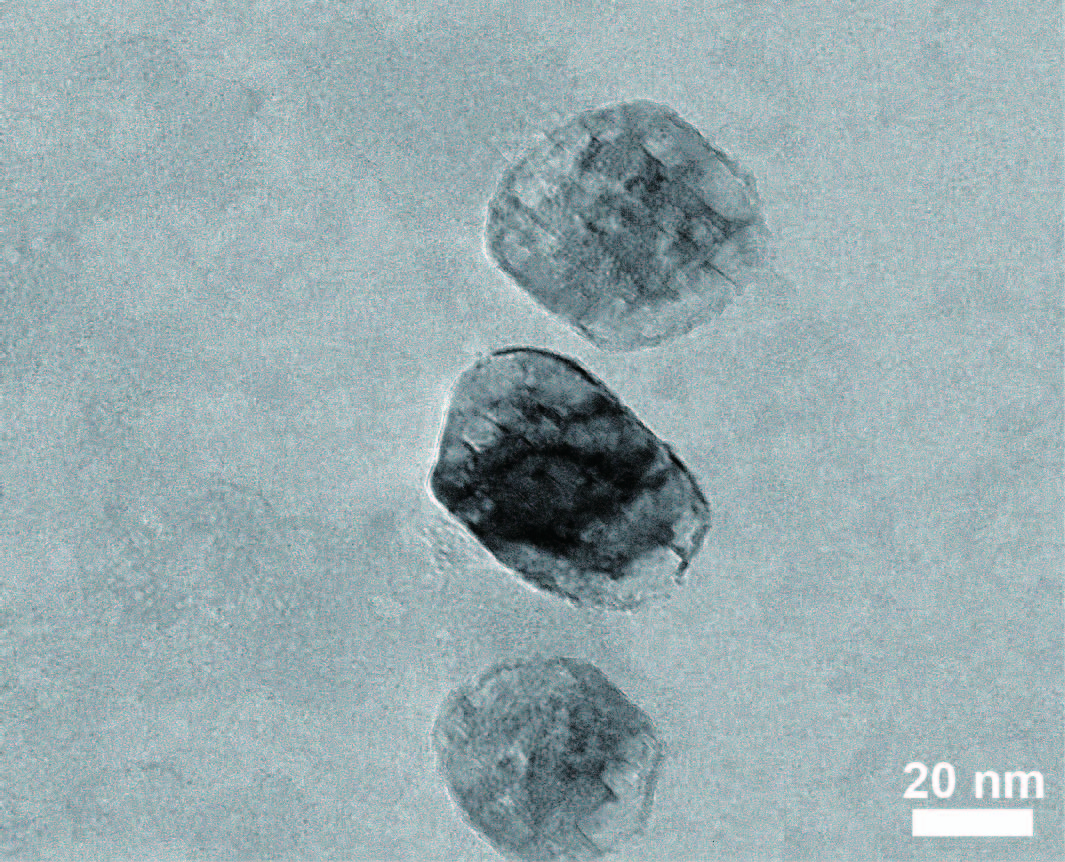


**Figure S1.** Representative TEM image of PEG-MnMOF nanosystem (scale bar: 20 nm).


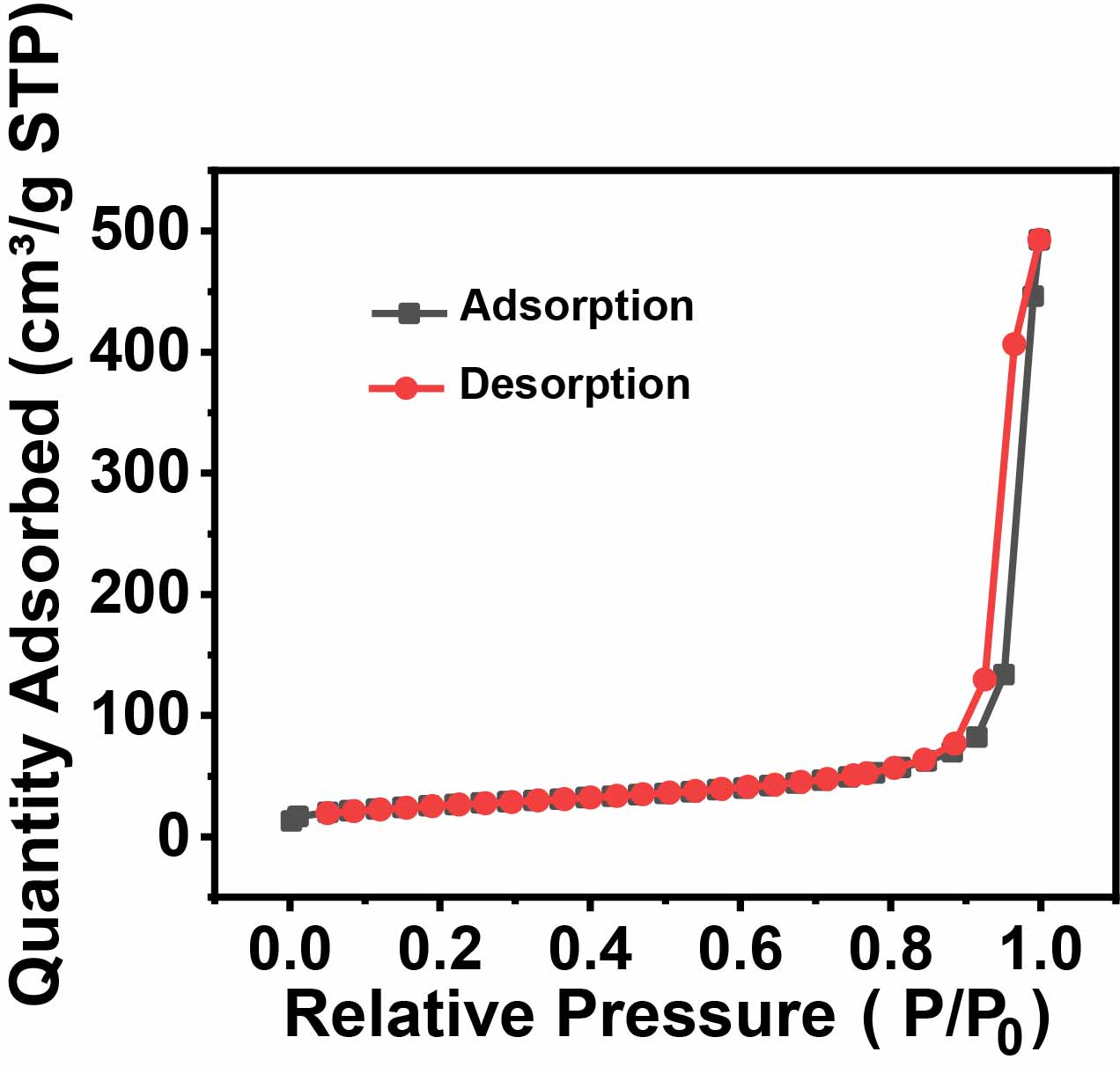


**Figure S2.** N_2_ adsorption-desorption isotherms of PEG-MnMOF.


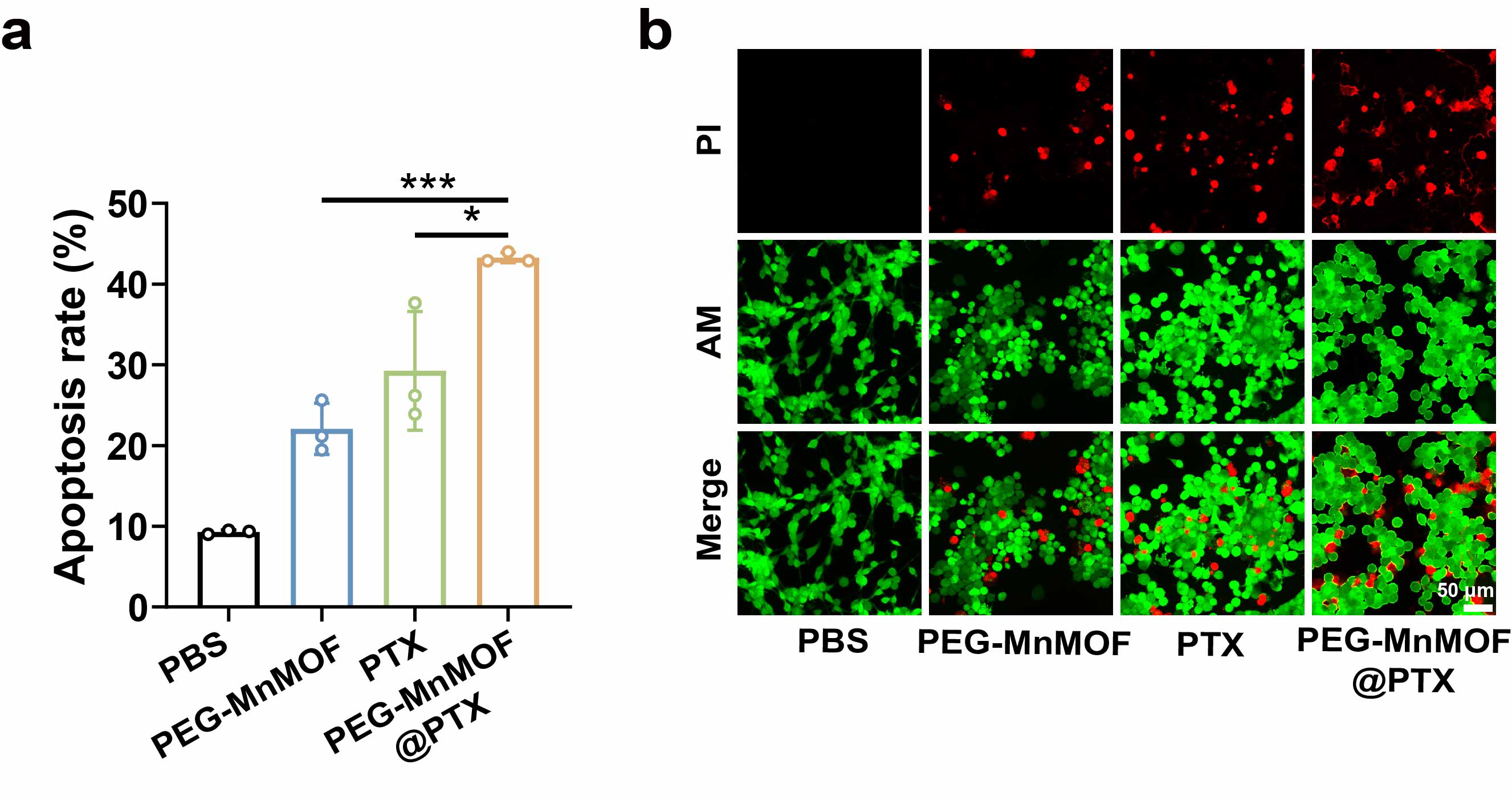


**Figure S3.** (a) Quantitative analysis of apoptosis rate (n = 3). (b) CLSM images of CT26 cells with different treatments and then stained with calcein AM (green, live cells) and PI (red, dead cells) (scale bar: 50 μm). Data are expressed as mean ± SD. Statistical significances were calculated *via* one-way ANOVA, *P<0.05, ***P<0.001.


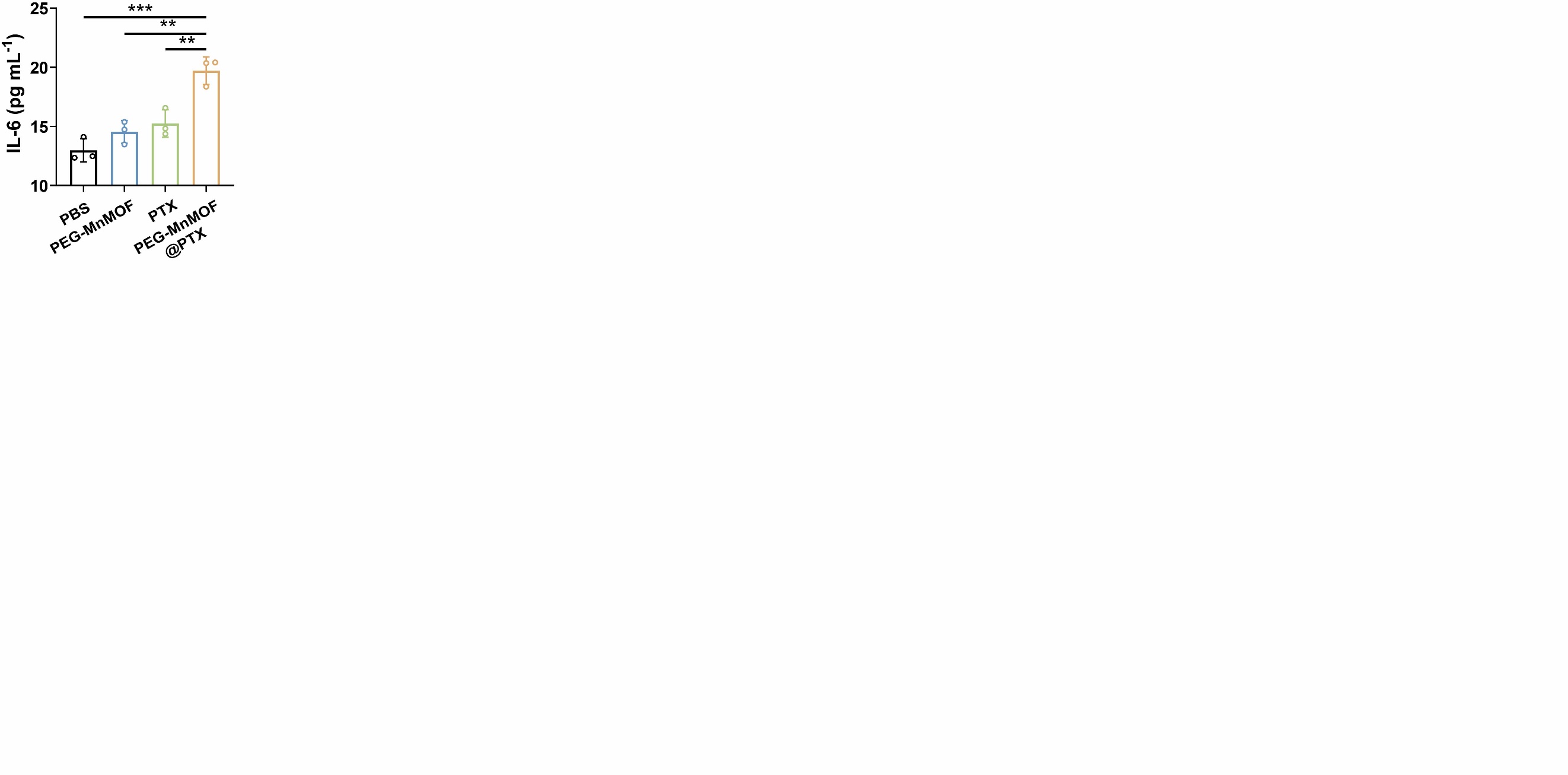


**Figure S4.** Cytokine levels of IL-6 in matured DCs suspensions (n = 3). Data are expressed as mean ± SD. Statistical significances were calculated *via* one-way ANOVA, **P<0.01, ***P<0.001.


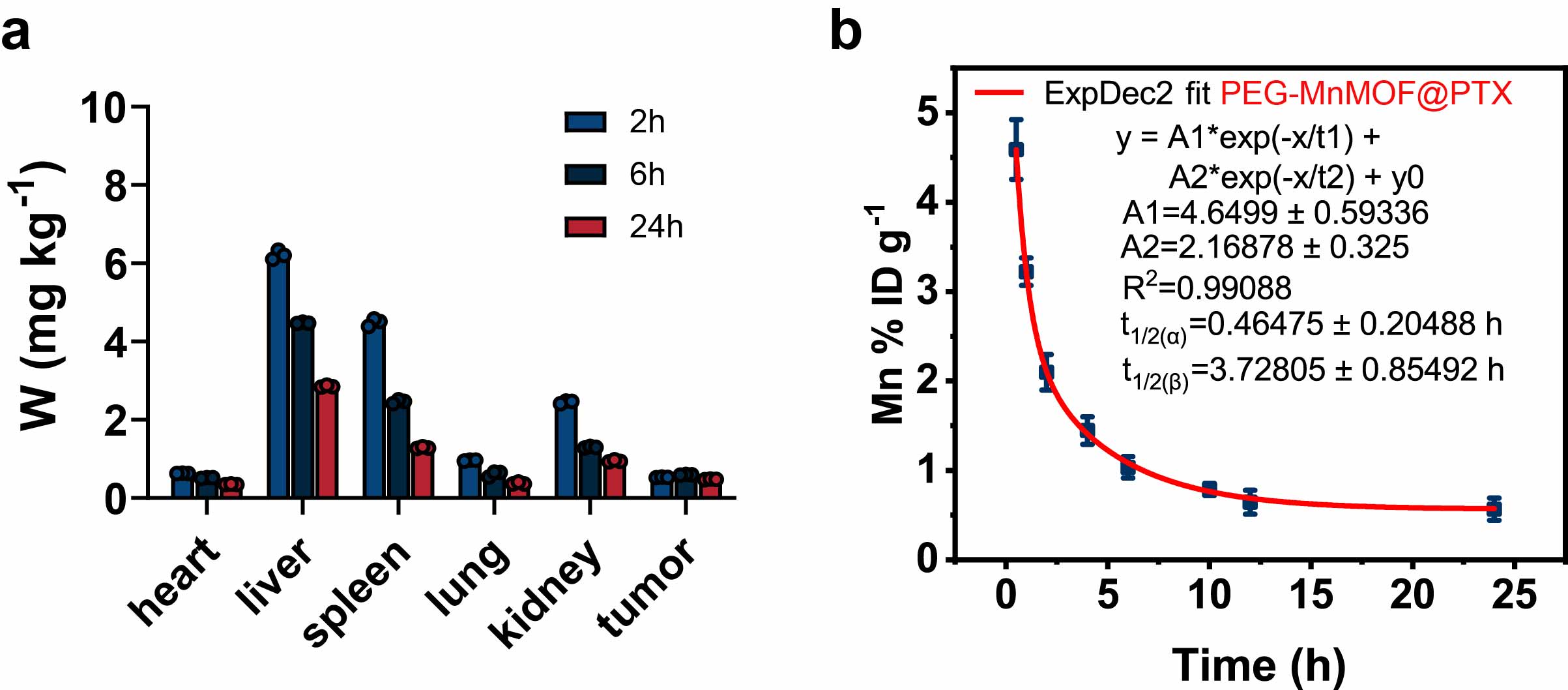


**Figure S5.** (a) Time-dependent biodistribution of Mn in major organs and tumors after *i.v.* injection of PEG-MnMOF@PTX (n = 3). (b) The blood-circulation time of administrated PEG-MnMOF@PTX is determined by ICP-OES (n = 3).


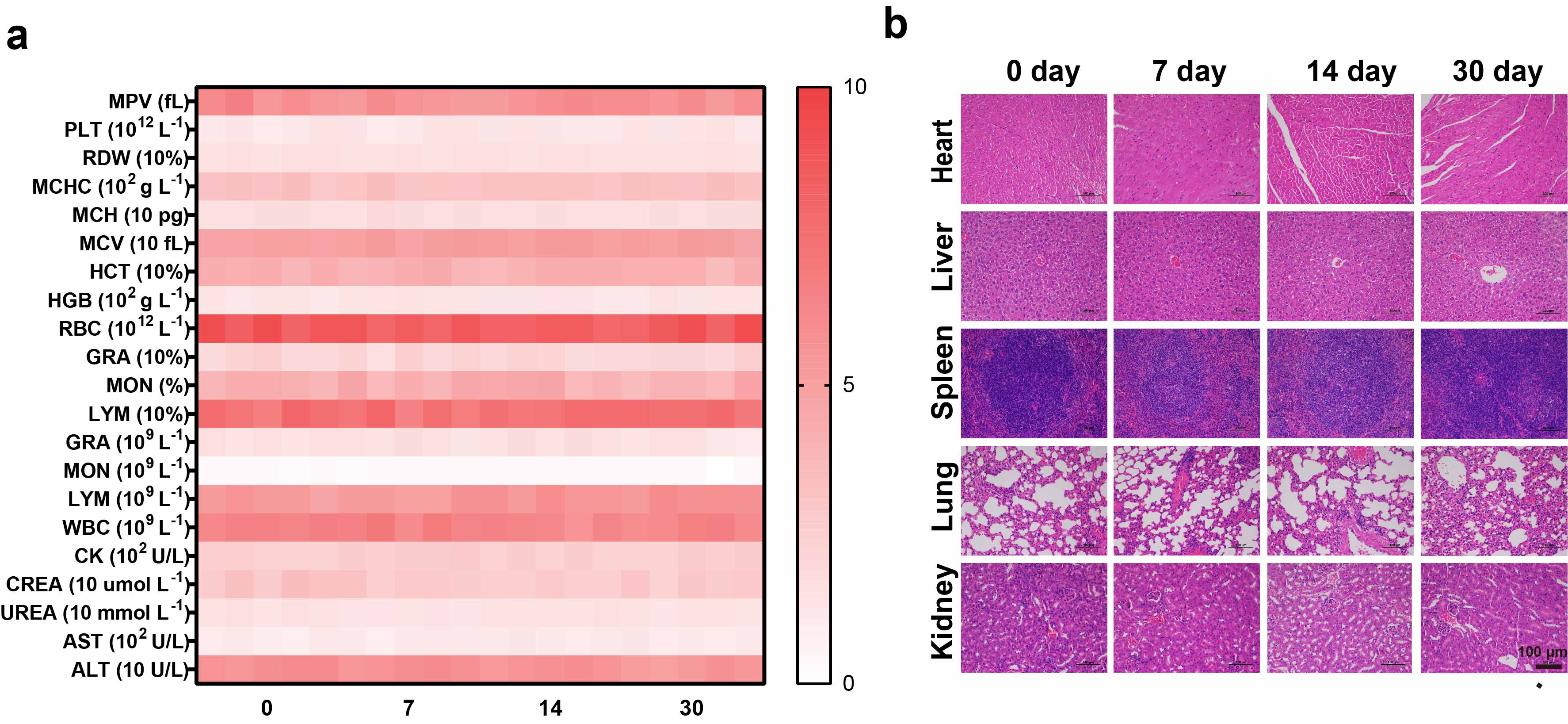


**Figure S6.** (a) Heatmaps for blood biochemistry and routine blood analysis. (b) H&E staining of major organs (heart, liver, spleen, lungs and kidneys) at different time points (on day 0, 7, 14, 30) (scale bar: 100 μm).


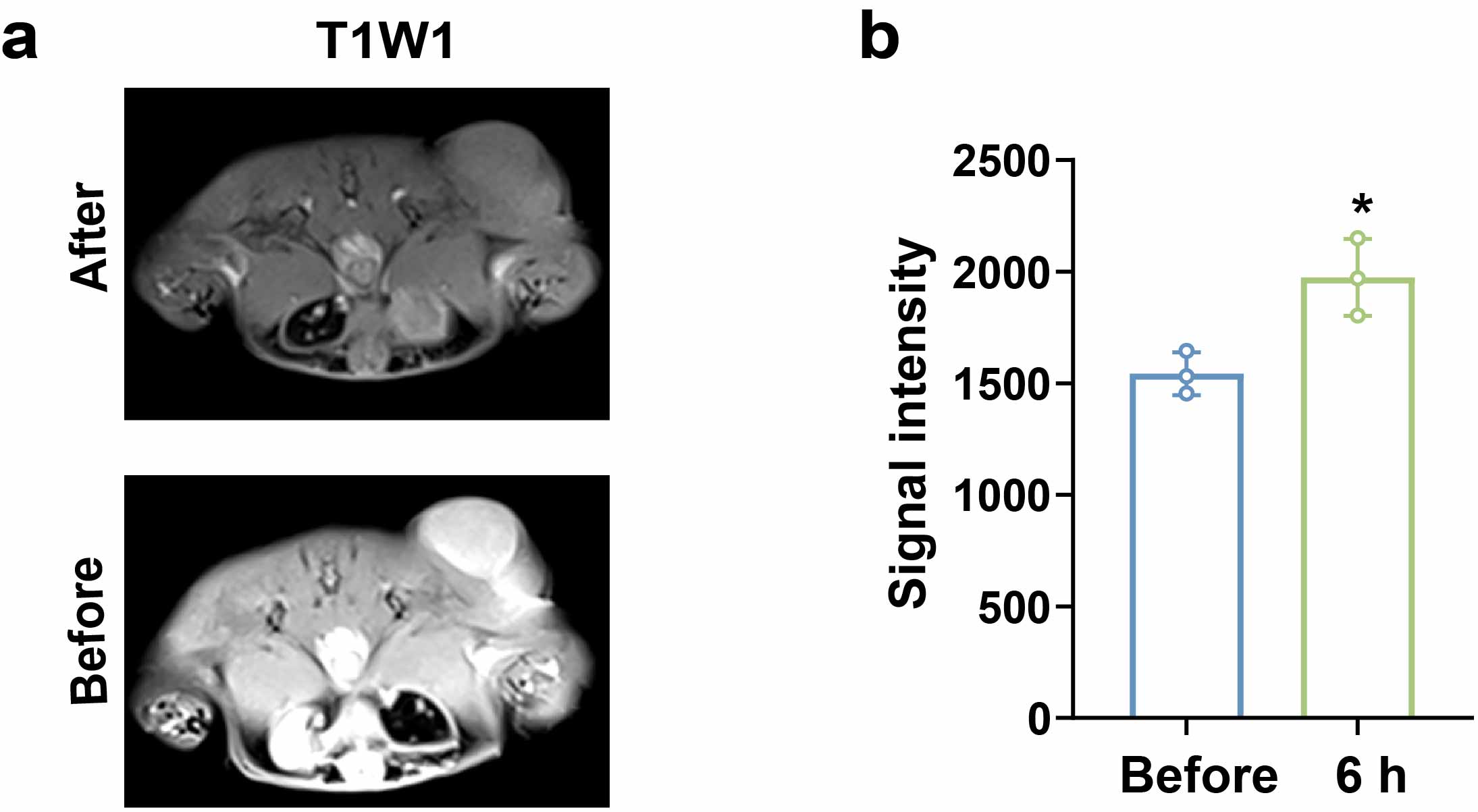


**Figure S7.** (a) T1-weighted MR images recorded at tumor region after intravenous injection of PEG-MnMOF@PTX at different time points and (b) corresponding quantitative analysis of signal intensity (n = 3). Data are expressed as mean ± SD. Statistical significances were calculated via one-way ANOVA, *P<0.05.


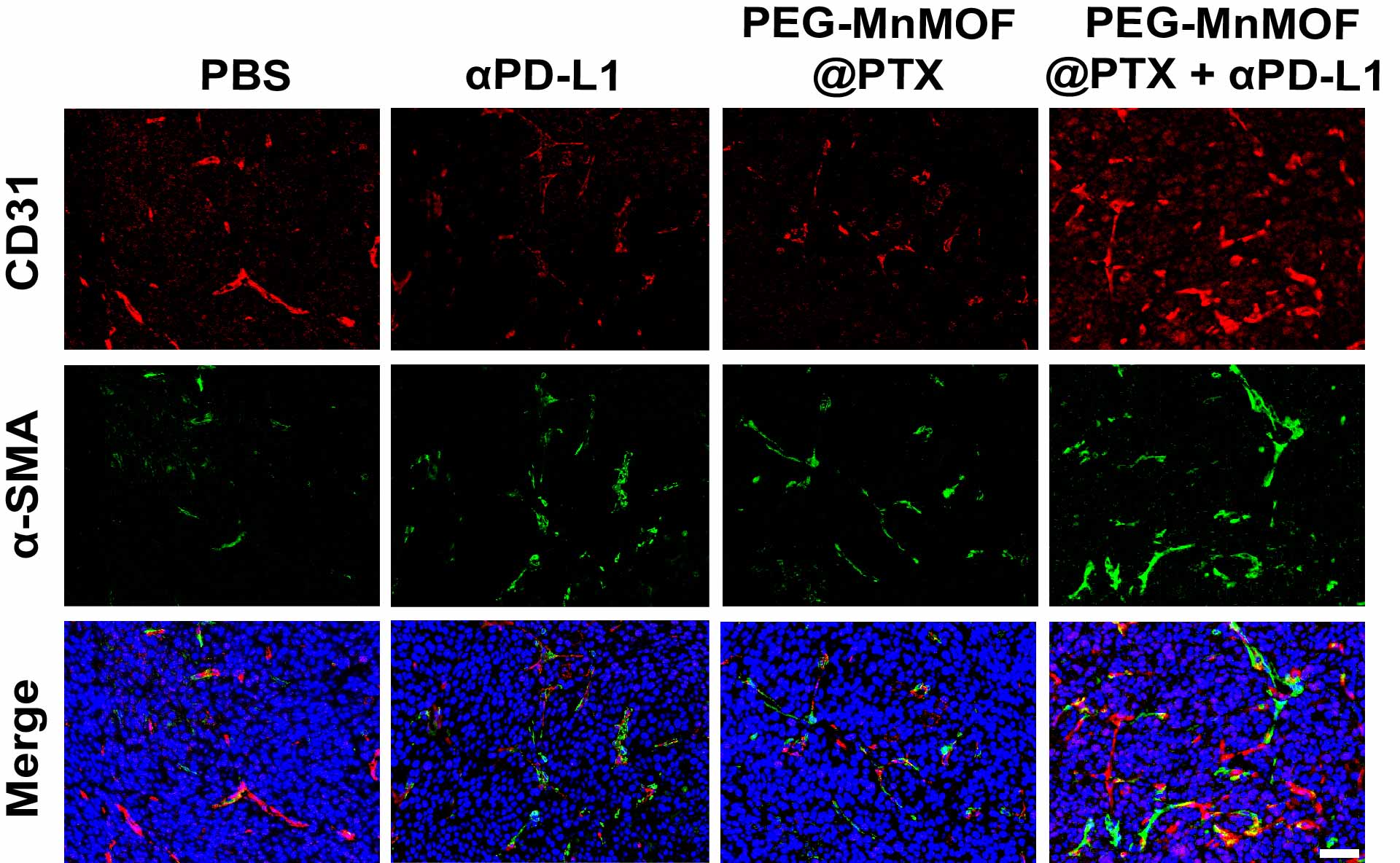


**50 μm**

**Figure S8.** Immunofluorescent images of tumor slices stained by DAPI (blue), anti-CD31 (red) and anti-αSMA (green) (scale bar: 50 μm).


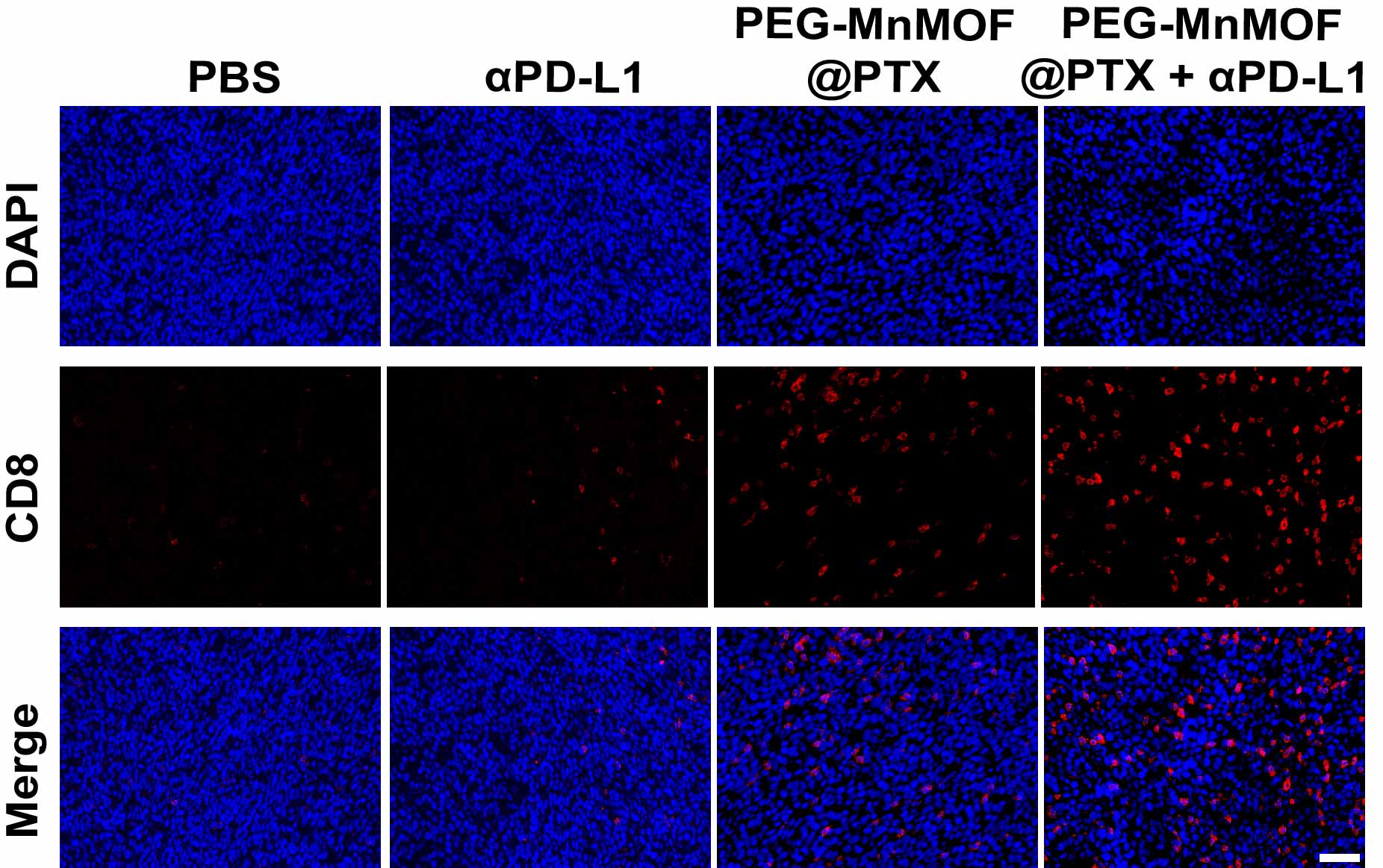


**50 μm**

**Figure S9.** Immunofluorescent images of tumor slices stained by DAPI (blue) and anti-CD8 (red) (scale bar: 50 μm).


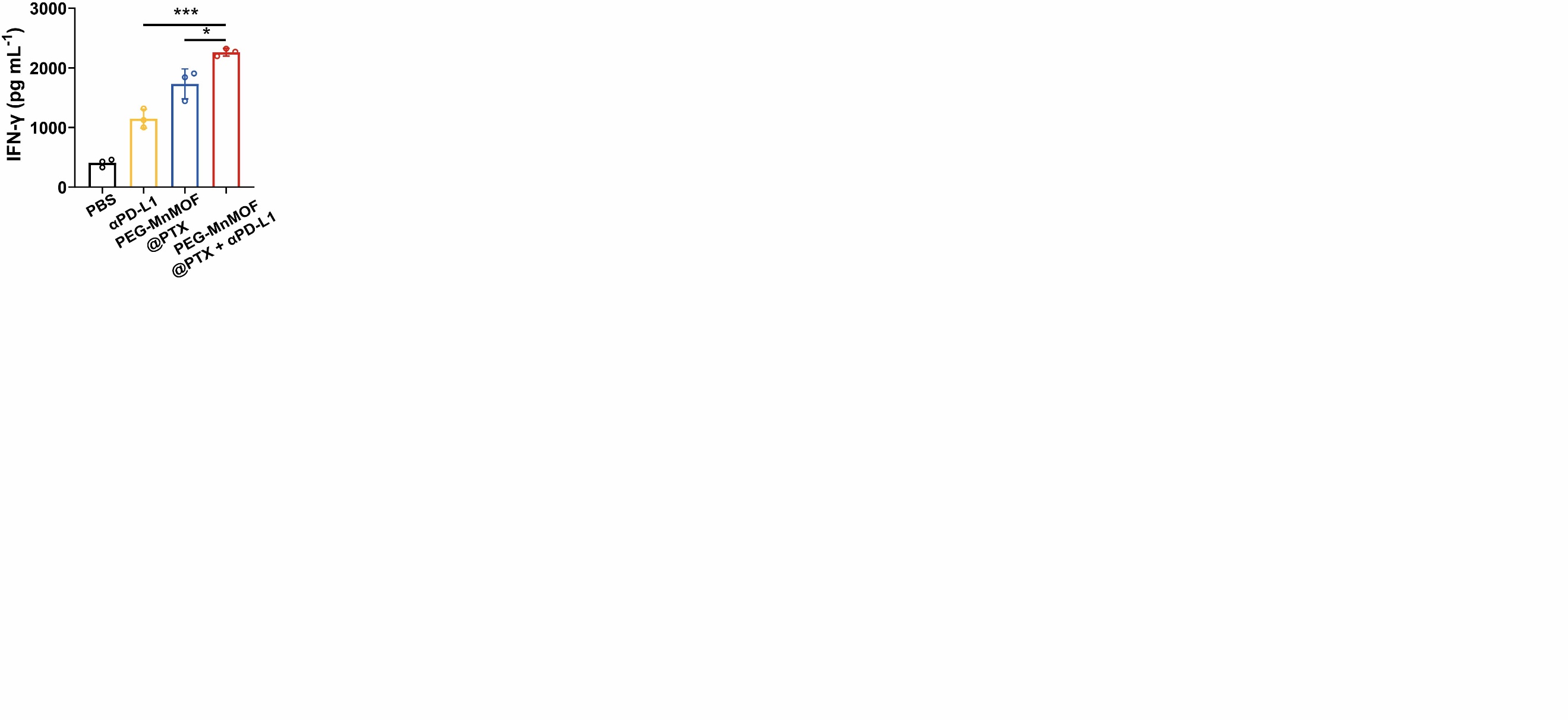


**Figure S10.** Serum levels of IFN-γ in CT26 tumor-bearing mice after different treatments (n = 3). Data are expressed as mean ± SD. Statistical significances were calculated *via* one-way ANOVA, *P<0.05, ***P<0.001.
